# Valproate reactivates HTLV-1 tax and reduces ABCB1/MDR1 expression in PBMCs derived from ATLL patients

**DOI:** 10.3389/fonc.2026.1721313

**Published:** 2026-03-12

**Authors:** Célima Mourouvin, Julie Tram, Laetitia Marty, Anika Marie-Delcasse, Gildas Belrose, Aneta Pluta, Raymond Césaire, Phillipe Hélias, Véronique Baccini, Jean-Marie Peloponese

**Affiliations:** 1 Université Montpellier (UM), Montpellier, France; 2 Institut de Recherche en Infectiologie de Montpellier (IRIM), Centre National de la Recherche Scientifique (CNRS), Montpellier, France; 3 Centre Hospitalier Universitaire de Martinique, Fort-de-France, Martinique; 4 Department of Virology and Viral Animal Diseases, National Veterinary Research Institute, Puławy, Poland; 5 Département de Radiothérapie-Oncologie-Hématologie, Centre Hospitalier Universitaire de La Guadeloupe, Pointe-à-Pitre, France; 6 Laboratoire d’Hématologie Centre Hospitalier Universitaire (CHU) de la Guadeloupe Pointe à Pitre Guadeloupe, Pointe-à-Pitre, France

**Keywords:** ABCB1/MDR-1, adult T-cell leukemia, chemoresistance, tax, valproate

## Abstract

Chemoresistance remains a major obstacle to effective treatment and durable remission in leukemia patients. Although initial responses to chemotherapy are often favorable, relapse frequently occurs due to the emergence of drug-resistant malignant clones. Resistance mechanisms may be intrinsic or acquired and involve drug efflux, impaired apoptosis, enhanced DNA repair, epigenetic alterations, dysregulated signaling pathways, and microenvironmental interactions. A central mediator of multidrug resistance is the ATP-binding cassette (ABC) transporter family, particularly ABCB1 (also known as P-glycoprotein or MDR-1), which actively exports chemotherapeutic agents such as etoposide, doxorubicin, and vincristine, thereby reducing intracellular drug accumulation. Adult T-cell Leukemia/Lymphoma (ATLL), an aggressive malignancy caused by Human T-cell Leukemia Virus type 1 (HTLV-1), is characterized by poor prognosis and marked resistance to chemotherapy. Despite the recent approval of novel therapeutic agents, treatment outcomes remain unsatisfactory, largely due to both inherent and acquired chemoresistance. Overexpression of ABCB1 has been identified as a key mechanism contributing to multidrug resistance in ATLL. We compared the expression profiles of ABC transporter genes in CD8⁺-depleted peripheral blood mononuclear cells (PBMCs) from HTLV-1 asymptomatic carriers and patients with acute ATLL. To investigate the role of the viral transactivator Tax in regulating ABCB1 expression, we used HuT78 and JPX9 T-cell lines. Furthermore, Tax expression was reactivated in CD8⁺-depleted PBMCs from acute ATLL patients using valproic acid, and subsequent changes in ABCB1 expression and chemosensitivity to etoposide and doxorubicin were assessed. We found that ABCB1 expression was significantly upregulated in CD8⁺-depleted PBMCs from patients with acute ATLL compared to asymptomatic HTLV-1 carriers. In contrast, expression of the viral protein Tax in HuT78 and JPX9 cell lines resulted in decreased ABCB1 levels. Reactivation of Tax expression using valproic acid in primary ATLL samples confirmed that Tax downregulates ABCB1 expression. Importantly, Tax reactivation restored sensitivity of ATLL cells to chemotherapeutic agents, including etoposide and doxorubicin. Our findings identify ABCB1 overexpression as a major contributor to chemoresistance in acute ATLL and demonstrate that the viral protein Tax negatively regulates ABCB1 expression. These results suggest that reactivation of Tax may reduce drug efflux capacity and restore chemosensitivity in resistant ATLL cells. Collectively, this study provides a rationale for exploring a “Tax-based shock-andkill” strategy as a potential therapeutic approach to overcome chemoresistance in ATLL.

## 1 Introduction

One of the major obstacles in oncology is the suboptimal efficacy of standard chemotherapy, which frequently stems from the interplay of multiple, interconnected chemoresistance mechanisms (MOCs) within tumor cells (1, 2). These mechanisms are linked to the resistome, a collection of drug-resistance genes that frequently undermines the success of pharmacological interventions (3). One of the most prominent mechanisms of chemoresistance is the reduced intracellular accumulation of active anticancer agents, which can result from either impaired drug uptake or enhanced drug efflux (1, 3, 4). Because the cytotoxic efficacy of most chemotherapeutics depends on their intracellular activity—often targeting essential processes such as DNA replication or mitosis—this reduction in cellular drug levels severely undermines treatment efficacy (5). Beyond transport‐related resistance, tumors may also evade therapy through altered drug metabolism, mutations or modifications of drug targets, upregulated DNA damage repair pathways, dysregulated apoptosis, remodeling of the tumor microenvironment, and shifts in cellular phenotype (3–5).

Human T-cell leukemia virus type 1 (HTLV-1) infects an estimated 5–10 million people worldwide, with endemic foci in Japan, the Caribbean, sub-Saharan Africa, and South America (6, 7). Transmission occurs via breastfeeding, sexual contact, and blood transfusions, primarily targeting CD4+ T cells (8–10). Although the vast majority of carriers remain asymptomatic, 2–5% progress to severe disorders—most prominently HTLV-1 Associated Myelopathy/Tropical Spastic Paraparesis (HAM/TSP) or Adult T-cell Leukemia/Lymphoma (ATLL) (8–10).

HTLV-1 integration into the host genome leads to chronic T-cell proliferation. The viral protein Tax plays a key role in this transformation, activating NF-κB, AP-1, and CREB/ATF signaling pathways, thereby promoting survival and proliferation (8–10). Another protein, HTLV-1 bZIP factor HBZ, is consistently expressed in ATLL and contributes to maintenance of the malignant phenotype by dampening host immune responses and promoting cell survival (11–13). ATLL itself is an aggressive and often treatment-resistant mature T-cell neoplasm, clinically divided into four subtypes: smoldering, chronic, acute, and lymphoma type (14, 15). Of these, the acute and lymphoma subtypes are particularly aggressive, frequently presenting with hypercalcemia, extensive organ infiltration, and a high leukemic burden. Despite intensive combination chemotherapy, median survival for patients with aggressive ATLL remains under one year (14, 15). In terms of treatment, acute forms of ATLL have shown limited responsiveness to standard chemotherapy regimens (e.g., CHOP14—cyclophosphamide, doxorubicin, vincristine, prednisone every 14 days; mLSG15—modified Lymphoma Study Group regimen 15), with only slight improvements noted for indolent subtypes when using antiviral therapy (azidothymidine [AZT] + interferon-α) (16–21). Allogeneic stem cell transplantation remains the only potentially curative option, though it is limited by high rates of transplant-related mortality and relapses (21). Chemoresistance significantly contributes to treatment failure in ATLL, developing due to viral oncoproteins and changes in host molecular pathways, especially involving MDR1/P-glycoprotein, encoded by the ATP-binding cassette sub-family B member 1 [ABCB1] gene (22–25). ABCB1 acts as an ATP-driven efflux pump, reducing intracellular levels of chemotherapeutics such as doxorubicin and cisplatin (26–29). In bladder cancer, resistance to cisplatin has been linked to upregulation of ABCB1 driven by hypoxia-inducible factor 1α (HIF-1α), a transcription factor also associated with increased tumor aggressiveness and growth (30). In breast cancer, treatment with paclitaxel induces expression of early growth response protein 1 (Egr-1), which interacts with the ABCB1 promoter to enhance its transcription (31, 32). Similarly, in osteosarcoma, studies show that the Wnt/β-catenin signaling pathway contributes to ABCB1-mediated resistance to doxorubicin (33).

Here, we demonstrate that the expression of key ATLL “resistome” components— including the organic anion-transporting polypeptide 2B1 (SLCO2B1), ABCB1, ATP-binding cassette sub-family G member 2 (ABCG2), low-density lipoprotein receptor-related protein 1 (LRP1), and the glutathione S-transferases P1 (GSTP1) and theta 1 (GSTT1) — is upregulated in PBMCs from acute ATLL patients. We also observed that, while HBZ/JunD has little effect, the AP-1 complex Fra-2 (Fos-related antigen 2)/JunD, but not Tax or p65, can transactivate the promoter of ABCB1 in HuT78 or JPX9 cell lines. Given the potential of epigenetic therapies to downregulate ABCB1 in solid tumors, we treated CD8^+^-depleted PBMCs from acute ATLL patients with valproate, an HDAC inhibitor. This treatment led to reactivation of HTLV-1 Tax, a marked reduction in ABCB1/MDR1 mRNA expression, and restored the sensitivity of ATLL cells to etoposide and doxorubicin. These findings suggest that epigenetic agents, such as valproate, may offer a novel strategy to reduce tumor burden and overcome chemoresistance in ATLL.

## 2 Materials and methods

### 2.1 HTLV-1 patients

The cohort comprised non‐infected controls, HTLV‐1–infected asymptomatic carriers (AC), and patients with acute ATLL (ATLL). Samples were obtained from the University Hospital of Fort‐de‐France (Martinique) and the University Hospital of Pointe‐à‐Pitre (Guadeloupe) in accordance with French bioethics regulations. ATLL diagnoses were established based on clinical presentation, hematological parameters, detection of the HTLV‐1 provirus in leukemic cells, and anti-HTLV‐1 serology, and cases were subclassified according to the JLSG criteria (14). Peripheral blood and serum specimens were provided by the Centre de Ressources Biologiques of Martinique (CeRBiM, CHU Martinique) and the Centre de Ressources Biologiques of Guadeloupe (KaruBioTech, CHU Guadeloupe). All study protocols involving human samples were approved by the Comité de Protection des Personnes.

### 2.2 Materials

pMDR1-1202 (Addgene, Cambridge, MA, USA) was a gift from Kathleen Scotto. pcDNA 3.1-HA-Fra-2 (GenScript, Piscataway, NJ, USA) was purchased from Genescript. pcAG-Flag-P65 and pEF-P52 were gifts from Junichiro Yasunaga (34). pCR3-SP1-WT, pcAG-Tax-Flag, pCMV-Tax M22, pCMV-Tax M47, pcDNA3.1 HBZ-Myc, and pcDNA 3.1 JunD-Flag were previously described (35, 36).

### 2.3 Cell culture and transfection

Human T-cell lines Jurkat (ATCC TIB-152, Manassas, VA, USA), HuT78 (ATCC TIB-161, Manassas, VA, USA), JPX9 (RRID: CVCL_0D86, BioVector NTCC, Beijing, China), HuT-102 (ATCC TIB-162, Manassas, VA, USA), C8166 (ECACC 88051601, Salisbury, UK), and ATL-2 (-) (CVCL_A6TF, RIKEN BioResource Center, Japan) were cultured in RPMI-1640 supplemented with 10% fetal calf serum (FCS) in low-binding T75 flasks (Sarstedt AG & Co, Nürmbrecht, Germany). HuT78 cells were transfected by electroporation using the Biorad Gene Pulser X following the manufacturer ‘s protocol. HEK 293T cells (ATCC CRL-3216, Manassas, VA, USA) were maintained in DMEM with 10% FCS and transfected using PEI MAX (1 mg/mL; Polysciences, Warrington, PA, USA) according to the supplier’s instructions. CD8(+)-depleted PBMCs from asymptomatic carriers (AC) and acute ATLL patients were isolated and cultured as previously described (16). Briefly, PBMCs from AC and ATLL patients were isolated from EDTA-anticoagulated blood samples by Ficoll-density gradient centrifugation and washed in phosphate-buffered saline (PBS). CD8+ cells were removed using anti-CD8 paramagnetic microbeads (Miltenyi Biotec, Paris, France) according to the manufacturer’s instructions. The resulting CD8+–depleted PBMCs were seeded at 1 × 10^6^ cells/mL in low-binding, round-bottom 24-well plates (Sarstedt AG & Co, Nürmbrecht, Germany) and cultivated in RPMI 1640 supplemented with 10% fetal calf serum (FCS), 2 mM glutamine, 100 IU/mL penicillin, and 100 µg/mL streptomycin (Eurobio, Courtaboeuf, France). Recombinant human IL-2 (hIL-2, STEMCELL Technologies SARL, Grenoble, France) was added at 10 ng/mL only for AC-derived cultures to support T-cell survival/expansion. ATLL patient-derived CD8-depleted PBMCs were cultured without exogenous IL-2 to avoid introducing additional activation/proliferation signals that could confound drug-sensitivity and gene-expression readouts.

### 2.4 Cell viability assay

Etoposide, doxorubicin hydrochloride, vinblastine sulfate, oxaliplatin, and camptothecin were purchased from Sigma-Aldrich (Courtaboeuf, France). Jurkat and ATL-2 cells were exposed to increasing concentrations of each drug for 48 hours. CD8+–depleted PBMCs from ATLL patients were cultured as described (12) and, were indicated, pretreated with 5 mM valproate (2-n-propylpentanoic acid; Sigma-Aldrich) for 24 hours prior to a 48-hour incubation with etoposide or doxorubicin. Cell viability was measured using PrestoBlue viability reagent (Thermo Fisher, Waltham, MA, USA) according to the manufacturer’s instructions. Fluorescence was recorded on a Tecan Spark10M microplate reader, and cell survival was assessed relative to untreated controls.

### 2.5 RNA analysis

Cells were collected and cryopreserved as dry pellets until use. Total nucleic acids were extracted using the Qiagen AllPrep DNA/RNA Mini Kit (Qiagen, Courtaboeuf, France) according to the manufacturer’s protocol. First-strand cDNA was synthesized from each RNA sample using the All-In-One 5X RT MasterMix (Applied Biological Materials Inc., Richmond, Canada). Quantification of ABC transporter transcripts was performed by real-time quantitative PCR (qPCR) with SYBR Green I Master Mix and gene-specific primer sets (**Supplementary Table 1**). For each primer pair, standard curves were generated on each plate using serial dilutions of a representative sample. Reactions were run in triplicate, and data collection and analysis were conducted with LightCycler 480 Software (Roche Diagnostics, Meylan, France). Relative expression levels were determined by the 2−ΔΔCT method (17), using HPRT1 as the internal reference gene, as previously described (12).

### 2.6 Western blotting

The primary antibodies used included: anti-Flag M2 (F3165), anti-Myc clone 9E10 (M5546), anti-HA (H3663), and anti-β-actin (A3853), all purchased from Sigma-Aldrich, France; anti-SP1 (ab12111) and HRP-conjugated anti-mouse secondary antibody (ab6789) from Abcam, Cambridge, UK; Nucloline (396400) from Thermo Fisher Scientific, Waltham, MA, USA; and anti-Tax-1 mAb clone 168 A51-2 (NIH AIDS Reagent Program) and anti-ABCB1/MDR-1 (D3H1Q) from Cell Signaling Technology, Danvers, MA, USA. Whole-cell lysates were prepared using RIPA buffer (10 mM Tris–HCl, pH 7.4; 150 mM NaCl; 1% NP-40; 1 mM EDTA; 0.1% SDS; 1 mM DTT), with or without boiling. Proteins were separated on 10% SDS-PAGE gels (Bio-Rad, Hercules, CA, USA) and transferred to PVDF membranes (Bio-Rad). Membranes were blocked and then incubated overnight at 4 °C with primary antibodies, followed by HRP-conjugated secondary antibodies (GE Healthcare, Chicago, IL, USA). Signal detection was performed using enhanced luminescence reagents (Roche, Basel, Switzerland) and imaged on a Chemidoc Imaging System (Bio-Rad).

### 2.7 Flow cytometry analysis of ABCB1 expression

HEK293T cells are transfected with 3 µg of either a control plasmid or a Flag-tagged Tax plasmid. Forty-eight hours after transfection, the cells are dissociated using FACS buffer (PBS 1X, 5 mM EDTA, 1% FCS) and counted (Invitrogen, C10228). A total of 2.5×10^5 cells per well are seeded into 96-well round-bottom plates and fixed for 10 minutes at room temperature with 10% formalin. After two washes in FACS buffer (2000 rpm for 3 minutes), cells were incubated for 2 hours at 4 °C with rabbit monoclonal anti-ABCB1 primary antibody (Cell Signaling, 12683) diluted 1:1000 in FACS buffer. Cells are washed twice more, then fixed again for 10 minutes with 10% formalin (Sigma-Aldrich, HT501128). For each condition, 1 × 10^5 cells were acquired on a Novocyte flow cytometer, and data were analyzed using Novocyte software.

### 2.8 Drug efflux assay

ABCB-1-mediated drug efflux was assessed using Calcein AM (37). HuT78 and ATL-2 cells (5 × 10^4 per well) were pre-incubated in Hank’s Balanced Salt Solution supplemented with 2% FCS and either 5 µM cyclosporin A (Sigma-Aldrich, 30024);, 100 µM verapamil hydrochloride (Sigma-Aldrich, V4629), or 0.25% DMSO as a vehicle control for 30 minutes. Cells were then incubated with 0.5 μM Calcein AM (Sigma-Aldrich) for 15 minutes at 37 °C. The intracellular calcein fluorescence was recorded for 8 minutes with excitation at 485 nm and emission at 538 nm on Tecan Spark10M microplate reader. Verapamil-treated samples served as the positive control for complete efflux inhibition (37).

### 2.9 Luciferase assay

Luciferase assays were performed as previously described (38). Briefly, cells were assayed on a Tecan Spark 10M microplate luminometer (Tecan) using the Genofax A and Genofax C kits (Yelen, Ensue la Redonne, France) following the manufacturer’s instructions. Firefly luciferase signals were normalized to Renilla luciferase activity to account for transfection efficiency.

### 2.10 ELISA analysis

Culture supernatants from CD8-depleted PBMCs were collected and assayed for HTLV-1 p19 antigen using a commercial enzyme-linked immunosorbent assay (ELISA) kit (Retrotek; Zeptometrix, Buffalo, NY), according to the manufacturer’s protocol. Absolute p19 concentrations were determined with a standard purified-antigen dilution curve.

### 2.11 *In vitro* proliferation – CFSE assay

CD8+-depleted PBMCs from ATLL patients were stained with 5 µM carboxyfluorescein diacetate succinimidyl ester (CFSE; Invitrogen, Life Technologies, Illkirch, France), according to the manufacturer’s instructions. Labeled cells were cultured for 5 days, then harvested, washed twice with 1x PBS, and fixed in 4% paraformaldehyde in PBS. CFSE dilution was analysed on FACSCanto II flow cytometer, and data were processed with ModFit LT 4.0 software (Verity Software House, Topsham, ME, USA). Dead cells and debris were excluded by gating, and propidium iodide (PI) staining distinguished live from dead cells. Proliferation was quantified by calculating the proliferation index (the average number of cell divisions per original cell) and the non-proliferative fraction (the percentage of cells that did not divide).

### 2.12 Statistical analyses

Comparisons of paired and unpaired data were performed using a one-way ANOVA with Dunn’s *post hoc* or the Mann-Whitney U test, as appropriate, in GraphPad Prism 8. Error bars represent the mean ± SEM. Differences were considered significant at **P<0.01 and ***P<0.001.

## 3 Results

### 3.1 ATLL intrinsic resistance to topoisomerase II inhibitors

**Figure 1:**
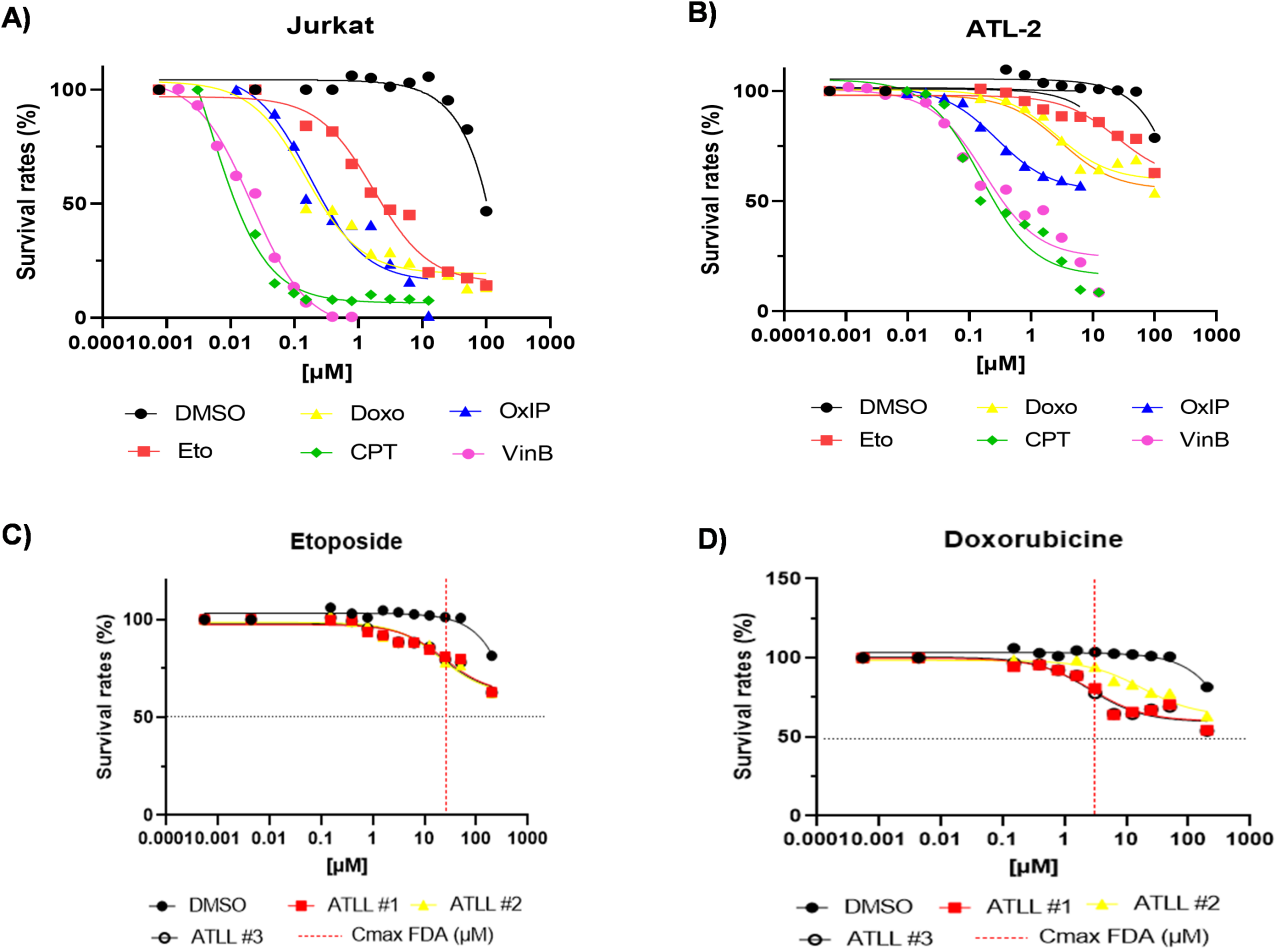
ATLL cells from untreated patients are intrinsically resistant to topoisomerase II inhibitors. **(A, B)** Jurkat and ATL-2 cell lines were treated for 48 hours with increasing concentrations of DMSO, etoposide (Eto), doxorubicin (Doxo), vinblastine (VinB), oxaliplatin (OxIP), and camptothecin (CPT). **(C, D)** CD8^+^ cell–depleted PBMC were incubated for 48 hours with increasing concentrations of Eto or Doxo, respectively. Cell viability was assessed using PrestoBlue vital dye (Invitrogen), and survival rate was calculated relative to untreated controls.

Adult T-cell leukemia/lymphoma (ATLL) is an aggressive cancer with a poor prognosis and limited response to standard treatments. To investigate the basis of this chemoresistance, we compared both the potency (half-maximal inhibitory concentration; IC50) and the combined efficacy and potency (area under the concentration–response curve, AUC) of five cytotoxic agents: doxorubicin (Doxo), etoposide (Eto), vinblastine (VinB), oxaliplatin (OxyP), and camptothecin (CPT) in HTLV-1–negative Jurkat cells versus an ATLL-derived cell line ATL-2 (**Figure 1**). Of these drugs, Doxo, Eto, VinB, and OxyP all exhibited significantly higher IC_50_ values (p ≤ 0.001) and reduced AUCs in ATL-2 compared to Jurkat cells (**Figures 1A, B**). Furthermore, Eto and Doxo showed minimal cytotoxicity in CD8+–depleted PBMCs isolated from three treatment-naïve patients with acute ATLL (**Figures 1C, D**). Collectively, these data (**Table 1**) strongly indicate that ATLL cells harbor intrinsic resistance to the topoisomerase II inhibitors etoposide and doxorubicin.

**Table 1:** Potency and the combined efficacy of chemotherapeutic agents against Jurkat and ATL-2 cell lines.

	Doxorubicin		Etoposide		VinBlastine		Oxiplatine		Campthotecin	
	Jurkat	ATL-2	Jurkat	ATL-2	Jurkat	ATL-2	Jurkat	ATL-2	Jurkat	ATL-2
Drug IC50 (µM)	0,1503	>100 #	1,718	>100 #	0,02128	0,1751	0,1735	>100 #	0,004761	0,1545
AUC (units)	1637 +/- 90,23	6501 +/-62,86 ****	2045 +/- 143,5	7643 +/- 70,94 ****	5,16 +/-0,2325	324 +/- 15,92 ***	235,3 +/- 17,59	386,4 +/- 5,91 *	103,9 +/-2,733	222+/- 3,45 ns

Jurkat and ATL-2 cells were cultured under low-binding conditions. Data are presented as mean ± standard deviation (n = 3). Significance values: p ≤ 0.05 (*), p ≤ 0.0001 (****). IC_50_ values were estimated from raw data where not directly calculated by GraphPad Prism.

### 3.2 ABCB1 upregulation drives etoposide and doxorubicin resistance in ATLL

**Figure 2:**
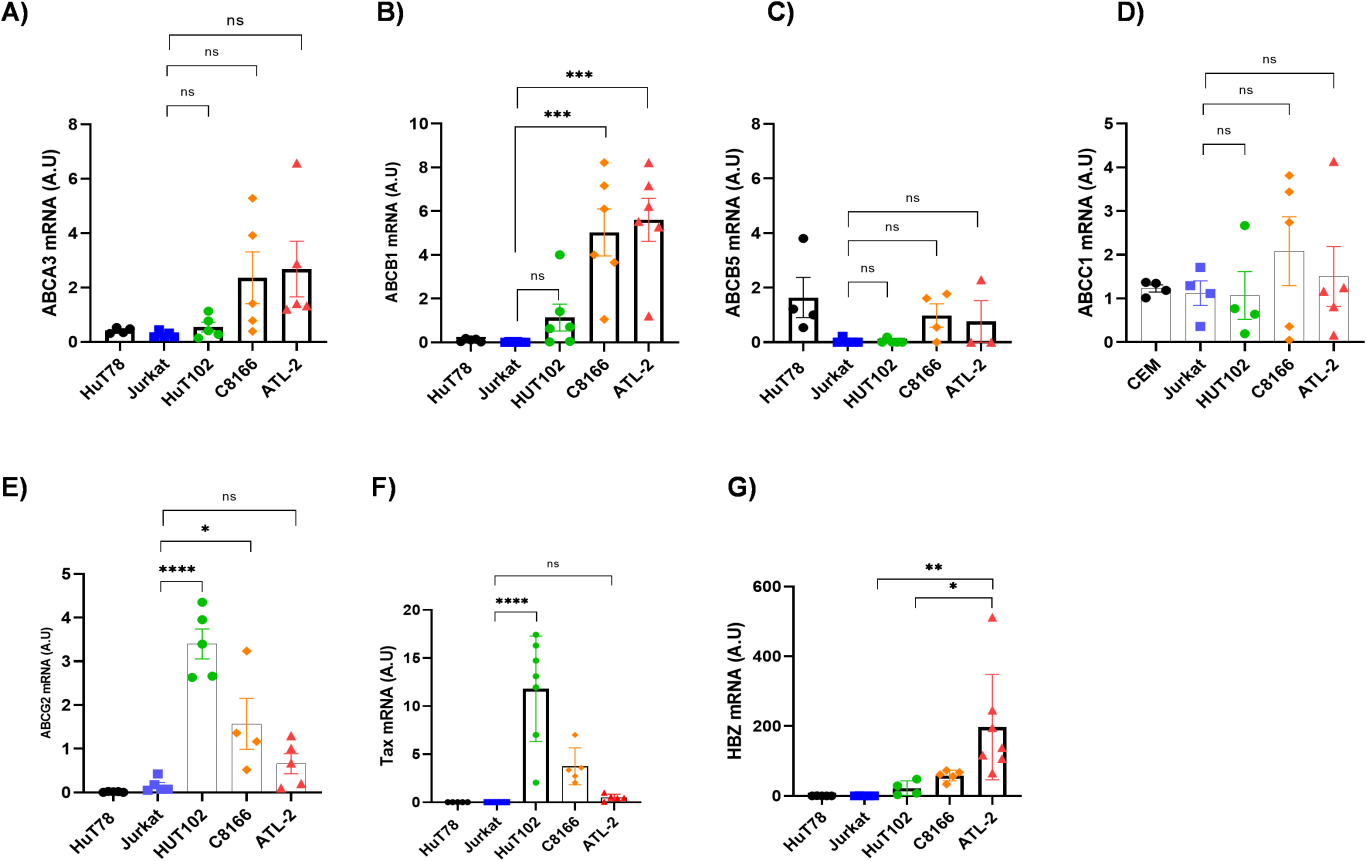
Expression analysis of efflux pumps from the ATP-ABC transporter family in HTLV-1-derived cell lines. **(A–E)** Expression of five ATP-ABC transporter family members associated with chemoresistance was assessed by RT-qPCR in two HTLV-1-negative cell lines (HuT78, Jurkat) and three HTLV-1-derived cell lines (HuT102, C81–66, ATL-2). **(F, G)** Relative expression of Tax and HBZ in HuT78, Jurkat, and HTLV-1-derived cell lines was measured by RT-PCR. Statistical significance was determined by one-way ANOVA with Dunn’s multiple comparisons post-test: ns, p ≤ 0.05, ** p ≤ 0.01; *** p ≤ 0.001, **** p ≤ 0.0001.

**Figure 3:**
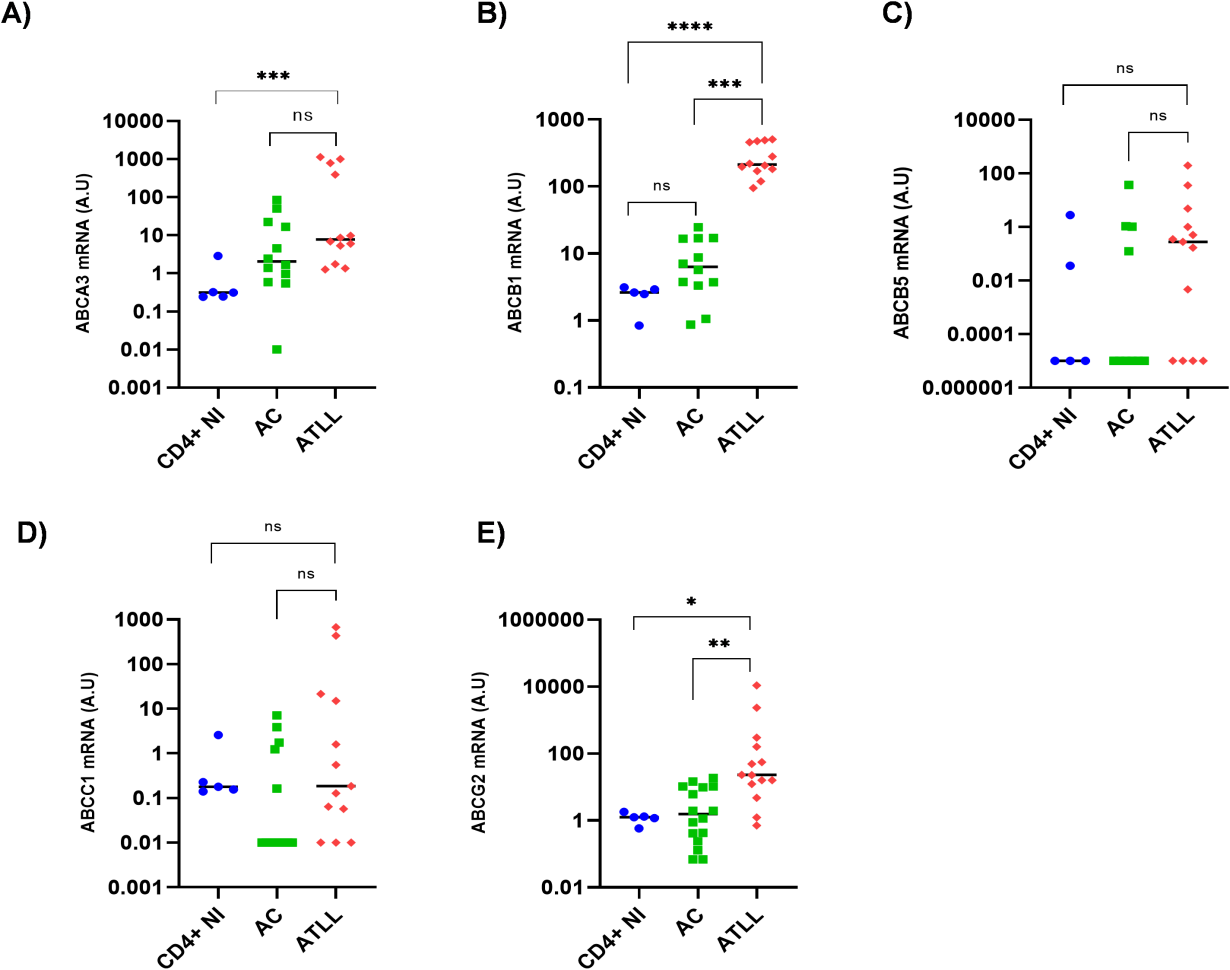
Analysis of efflux pump expression from the ATP-ABC transporter family in PBMCs from HTLV-1-infected patients. **(A–E)** Expression of five ATP-binding cassette (ABC) transporter genes (ABCA3, ABCB1, ABCB5, ABCC1, and ABCG2) was measured in CD4^+^ T cells from five non-infected donors (NI), twelve HTLV-1 asymptomatic carriers (AC), and thirteen untreated acute ATLL patients (ATL) by RT-qPCR. Statistical significance was determined using a one-way ANOVA test with Dunn’s multiple comparisons post-test; ns p ≤ 0.05, ** p ≤ 0.01; *** p ≤ 0.001, **** p ≤ 0.0001.

We investigated resistance to etoposide and doxorubicin in ATL-2 cell lines and in primary cells from ATLL patients. First, we examined the expression of five ATP-binding cassette transporters (ABCA3, ABCB1, ABCB5, ABCC1, and ABCG2) (**Figure 2**) (27); low-density lipoprotein receptor-related protein 1 (LRP1; also known as apolipoprotein E receptor, APOER, or CD91) (39); three solute carrier transporters involved in etoposide and doxorubicin uptake (SLCO2B1, SLC22A4, and SLC22A5) (40); and two glutathione S-transferases essential for chemotherapeutic detoxification (GSTP1 and GSTT1) (**Supplementary Figure 1**). These analyses were performed on T cell lines (**Figure 2**, **Supplementary Figure 1**) and CD8+–depleted PBMCs from untreated ATLL patients (**Figure 3**, **Supplementary Figure 2**). When comparing the expression of these ten genes in the ATL-2 resistant line versus control T-cell lines (HuT78 and Jurkat) (**Figures 2A-G**), only the ATP-dependent efflux pump ABCB1 was consistently upregulated in both the ATL-2 (**Figure 2B**) and CD8^+^-depleted cells from untreated ATLL patients (**Figure 3B**). ABCB1 (also known as MDR1) is an ATP-binding cassette (ABC) transporter that exports a wide range of chemotherapeutics and phospholipids across the plasma membrane, and its role in mediating drug resistance is well established (26–28). ABCB1 facilitates the transport and lysosomal sequestration of P-glycoprotein substrates—such as doxorubicin—thereby reducing their cytotoxic effects (26–28). Moreover, translational studies have shown that low-density lipoprotein receptor-related protein 1 (LRP-1) modulates ABCB1 expression and subcellular localization via endocytic pathways, thereby increasing Dox resistance (41). While no significant difference in LRP-1 mRNA levels was observed between the tested T-cell lines (**Supplementary Figure 1D**), we found a substantial increase in LRP-1 expression in untreated ATLL patients compared to non-infected individuals or HTLV-1 asymptomatic carriers (AC) (**Supplementary Figure 2D**). In contrast, SLCO2B1—which mediates etoposide uptake—was significantly overexpressed in patient PBMCs but remained unchanged in the ATL-2 cell line (**Supplementary Figures 1A**, **2A**).

### 3.3 ABCB1 overexpression and functional inhibition assays

**Figure 4:**
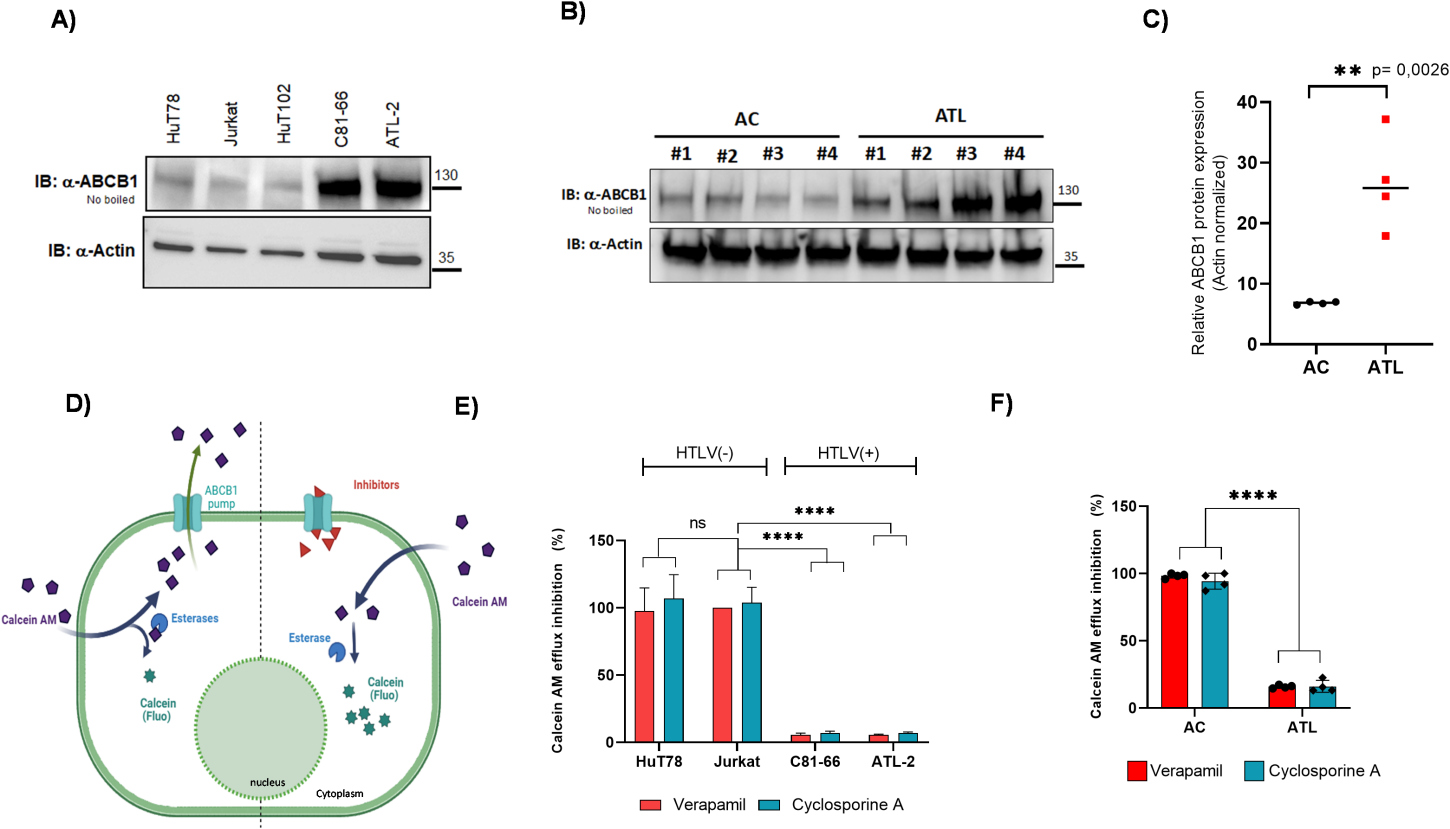
Verapamil does not inhibit ABCB1 in ATLL primary cells. **(A)** Western blot of unboiled extracts from control T-cell lines HuT78 (lane 1) and Jurkat (lane 2), and HTLV-1-infected lines HUT-102 (lane 3), C8166 (lane 4), ATL-2 (lane 5). IB, immunoblot. **(B)** Western blot of unboiled extracts from peripheral blood mononuclear cells of four HTLV-1 asymptomatic carriers (AC; lanes 1–4) and four untreated acute ATLL patients (ATL; lanes 5–8). IB, immunoblot. **(C)** Densitometric quantification of ABCB1 normalized to actin using ImageJ. Data are mean ± SD; significance by unpaired t-test (p = 0.0026). **(D)** Calcein-AM efflux assay schematic for measuring ABCB1 activity **(D)** in T-cell lines **(E)** and HTLV-1-infected PBMCs **(F)**. Inhibition is expressed relative to verapamil-treated controls. Data are mean ± SD; one-way ANOVA with Dunn’s multiple-comparisons post-test: ns, not significant; *p ≤ 0.05; **p ≤ 0.01; ***p ≤ 0.001; ****p ≤ 0.0001.

Elevated ABCB1 protein expression was clearly detected in ATL-2 cells and CD8-depleted PBMCs from untreated ATLL patients, as shown by immunoblotting (**Figures 4A, B**). This was further confirmed by normalizing the ABCB1 signal against actin (**Figure 4C**), highlighting the significance of ABCB1 upregulation in these patient samples. To counteract ABCB1-mediated drug resistance, we tested two known ABCB1-1 inhibitors: verapamil, a calcium channel blocker (42), and cyclosporin A (CsA), an immunosuppressant targeting calcineurin (43). We hypothesized that pre-treatment with either drug would inhibit ABCB1 efflux activity and restore ATLL cell sensitivity to Eto or Doxo. Consequently, T-cell lines and PBMCs from patients with acute ATLL were incubated with 100 µM verapamil or 5 µM CsA for 30 minutes, and transporter inhibition was assessed using the calcein AM assay. Calcein AM is a non-fluorescent, cell-permeable substrate that intracellular esterases convert into fluorescent calcein. Active ABCB1 rapidly exports calcein AM, whereas inhibitor treatment increases intracellular fluorescence, indicating pump blockade (37). Under verapamil treatment, ABCB1 activity is blocked, leading to increased intracellular calcein accumulation, which serves as a quantitative measure of transporter inhibition (37). However, neither verapamil nor CsA reduced ABCB1-mediated efflux in HTLV-1–infected cell lines (C81-66 and ATL-2) or CD8+-depleted PBMCs from ATLL patients. Both treatments failed to increase calcein retention in these cells compared to Jurkat controls (**Figure 4D**) or HTLV-1 asymptomatic carriers (AC) (**Figure 4E**), indicating that ABCB1 pump activity persisted.

### 3.4 Transcriptional regulation of ABCB1 by Tax, HBZ, and host transcription factors

**Figure 5:**
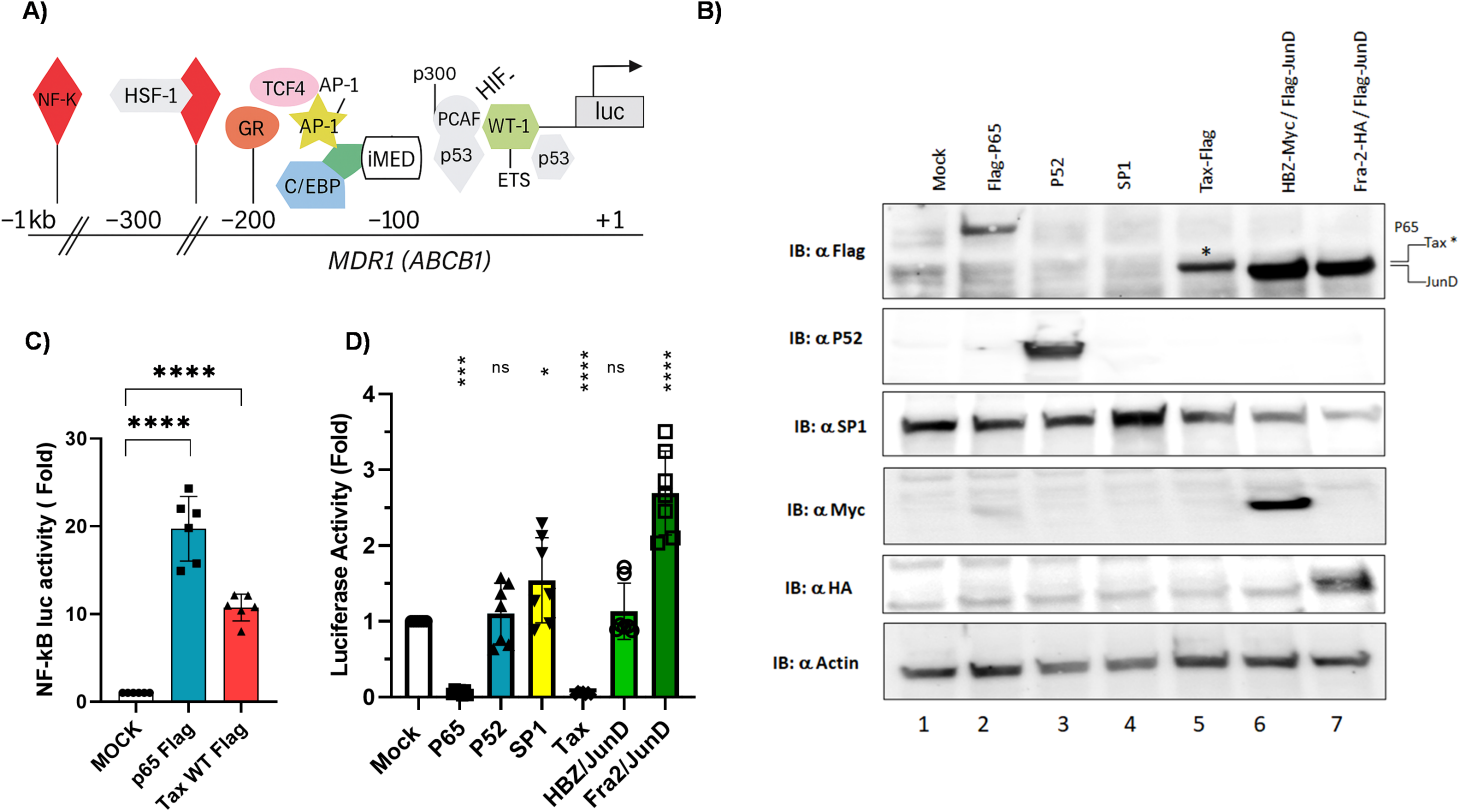
Tax inhibits ABCB1 transcription in HEK293T cells. **(A)** Schematic of transcription-factor binding sites in the proximal ABCB1 promoter. **(B)** Western blots confirming expression of P65-Flag, P52, SP1, Tax-Flag and JunD-Flag in the presence of HBZ-Myc or Fra-2-HA. Actine served as a loading control. **(C)** HEK293T cells were co-transfected with a firefly luciferase reporter under the control of an NF-κB–dependent promoter, together with expression vectors for P65-Flag and Tax-Flag. pRcActin-LacZ was included to normalize transfection efficiency. Luciferase activity was measured 48 h post-transfection and is shown as fold change relative to the mock control (set = 1), with mean ± SD from five independent experiments. **(D)** HEK293T cells were co-transfected with a firefly luciferase reporter driven by the ABCB1 promoter, together with expression vectors for P65-Flag, P52, SP1, Tax-Flag, JunD-Flag, and either HBZ-Myc or Fra-2-HA. pRcActin-LacZ was included to normalize transfection efficiency. Luciferase activity was measured 48 h post-transfection and is shown as fold change relative to the mock control (set = 1), with mean ± SD from seven independent experiments.

**Figure 6:**
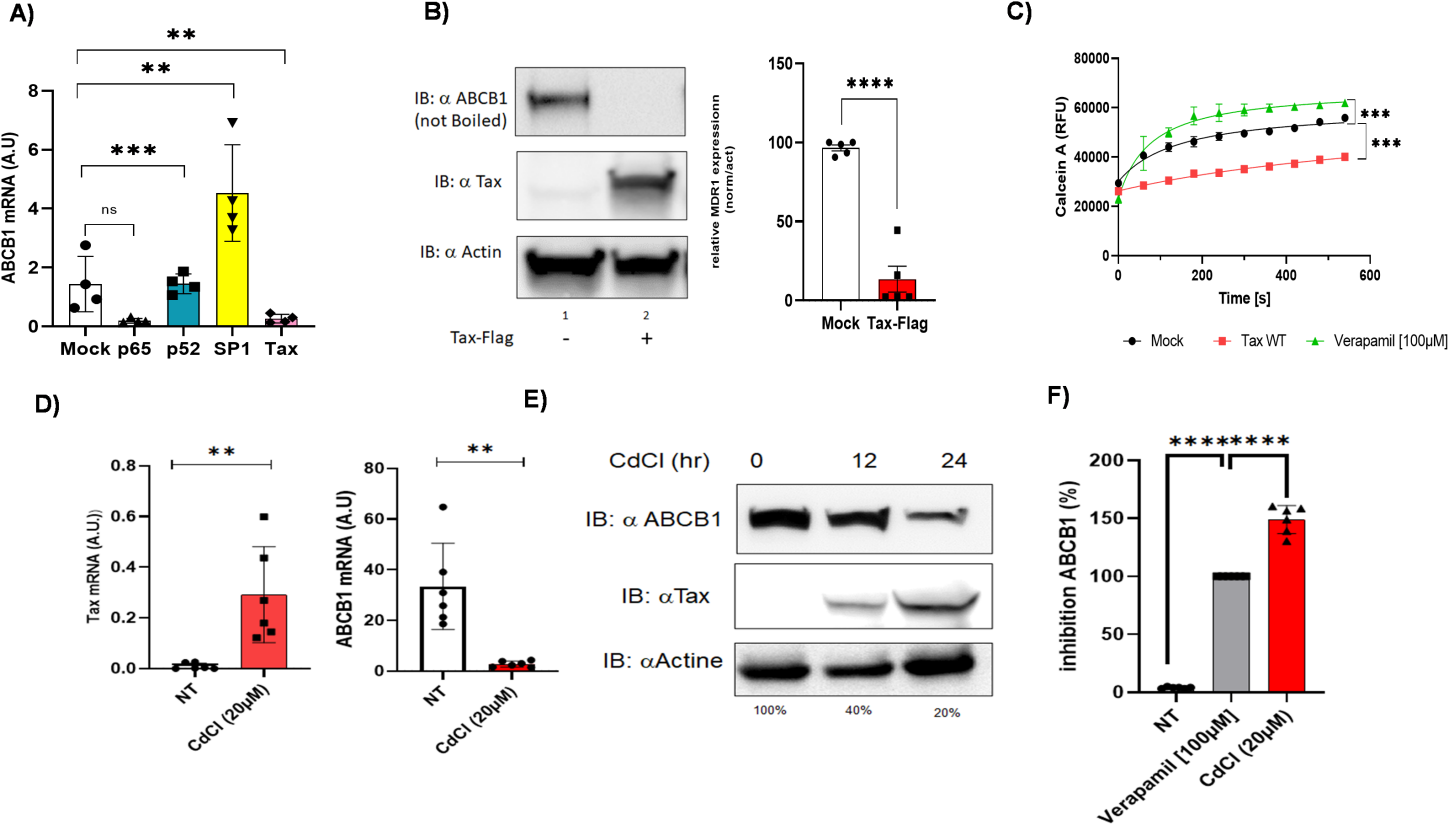
Tax inhibits ABCB1 expression in HuT78 and JPx9T-cell lines. **(A)** HuT78 cells were transfected with P65-Flag, P52, SP1, or Tax-Flag plasmids. Cells were harvested 48 hours post-transfection, and ABCB1 mRNA levels were measured by RT-qPCR. Statistical significance was determined by one-way ANOVA with Dunn’s multiple comparisons post-test (ns, p ≤ 0.05, * p ≤ 0.01, ** p ≤ 0.001, *** p ≤ 0.00001). **(B)** Western blot analysis of Tax-Flag and ABCB1 expression, with actin as the loading control. **(C)** Calcein-AM efflux kinetics measuring ABCB1 activity in Hut78 cells for 8 minutes with 100 µM verapamil (green curve) as a control. **(D-F)** JPX9 cells (human T-cell line with inducible HTLV-1 provirus) were treated for 24 hours with 20 µM cadmium chloride (CdCl2).Tax and ABCB1 expression was measured by RT-qPCR **(D)** and Western blotting **(E, F)** Inhibition of ABCB1 efflux activity was measured using the Calcein AM assay. The percentage of inhibition was calculated relative to control cells treated with verapamil.

**Figure 7:**
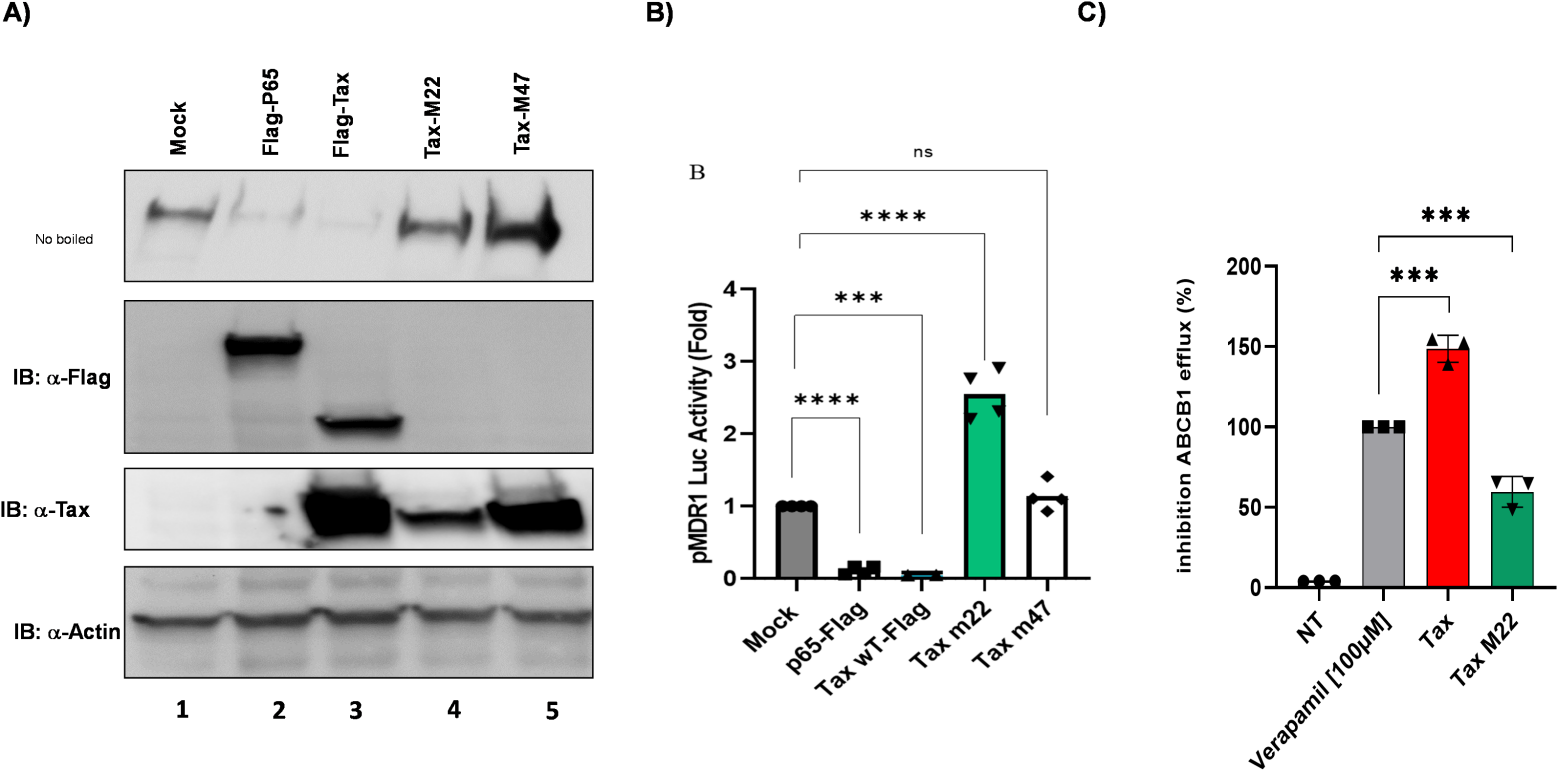
Tax inhibits ABCB1 expression through its NF-κB signaling. **(A)** Western blot analyses assessed the expression of P65-Flag, Tax-Flag, Tax M22, and Tax M47, with Actin shown as a loading control. **(B)** HEK293T cells were co-transfected with a plasmid carrying the firefly luciferase reporter gene under the control of the promoter of ABCB1, along with P65-Flag, Tax-WT Flag, and the Tax mutants Tax M22 (NF-κB deficient) and Tax M47 (CREB deficient) expression vectors, in addition to pRcActin-LacZ for normalization of transfection efficiency. Cells were harvested 48 h post-transfection and assayed for luciferase activity. The results show a fold increase over the mock control and represent the mean values from four independent experiments. **(C)** Calcein fluorescence accumulation was measured for 8 minutes in HEK293T cells transfected with Tax WT or Tax M22. The percentage of inhibition was assessed relative to that of verapamil-treated untransfected cells.

We hypothesized that HTLV-1-encoded proteins Tax and HBZ might regulate ABCB1 expression in ATLL cells. Tax is a potent transcriptional activator that recruits CREB/ATF family members to gene promoters (44). Meanwhile, HBZ has been shown to regulate transcription by forming a JunD-containing complex at the hTERT promoter (45). To explore the roles of Tax, HBZ, and other transcription factors in regulating ABCB1 transcription (**Figure 5A**) (46), we performed luciferase reporter assays in HEK293T cells and confirmed the expression of each transcription factor via western blotting (**Figure 5B**). Specifically, we compared the ability of Tax, HBZ/JunD, SP1, and Fra-2/JunD to activate the ABCB1 promoter. As expected, both p65 (the NF-κB subunit) and Tax strongly activated an NF-κB-dependent promoter (**Figure 5C**); however, both factors significantly decreased ABCB1 transcription (**Figure 5D**). Interestingly, the Fra-2/JunD heterodimer effectively activated transcription from the ABCB1 promoter. Tax is a major regulator of cellular gene transcription (17, 44), but paradoxically, it can also act as a repressor of gene expression (47–51). To determine whether this suppression is also observed for ABCB1, we transfected HuT78-cell lines with NF-κB p65, p52, SP1, and Tax. Tax significantly reduced ABCB1 mRNA levels (**Figure 6A**), protein expression (**Figure 6B**, **Supplementary Figure 3**), and ABCB1-mediated efflux activity (**Figure 6C**). Next, Tax expression was induced by cadmium chloride in JPX-9 T lymphocytes. Under these conditions, Tax RNA levels remained substantially lower than those observed in HTLV-1-infected HuT102 cells (**Figure 2F**). We also observed a 13.9-fold decrease in ABCB1 mRNA levels (**Figure 6D**), along with decreased ABCB1 protein levels (**Figure 6E**) and efflux activity (**Figure 6F**). Notably, Tax was absent in the tested ATLL cells, which showed high levels of ABCB1 transcription (shown in **Figures 2F**, **3F**). Tax is known to activate two major pathways: CREB and NF-κB (17, 44). To investigate whether Tax’s inhibitory effect on ABCB1 is linked to its activity on CREB or NF-κB, we compared two Tax mutants with well-documented phenotypes: M47 (defective in CREB activation) and M22 (defective in NF-κB activation) (52, 53), and evaluated their ability to regulate ABCB1 expression (**Figure 7**). HuT78 cells were co-transfected with an ABCB1-driven luciferase reporter and either NF-κB p65-Flag, Tax-Flag, Tax M22, or Tax M47. In these transfected cells, the Tax M22 mutant strongly activated ABCB1 transcription (**Figure 7A**) and increased efflux activity (**Figure 7C**). Overall, these findings suggest that reactivation of Tax could counteract MDR in ATLL cells by downregulating ABCB1 expression.

### 3.5 Valproate reactivates viral genes and suppresses ABCB1 in primary ATLL cells

**Figure 8:**
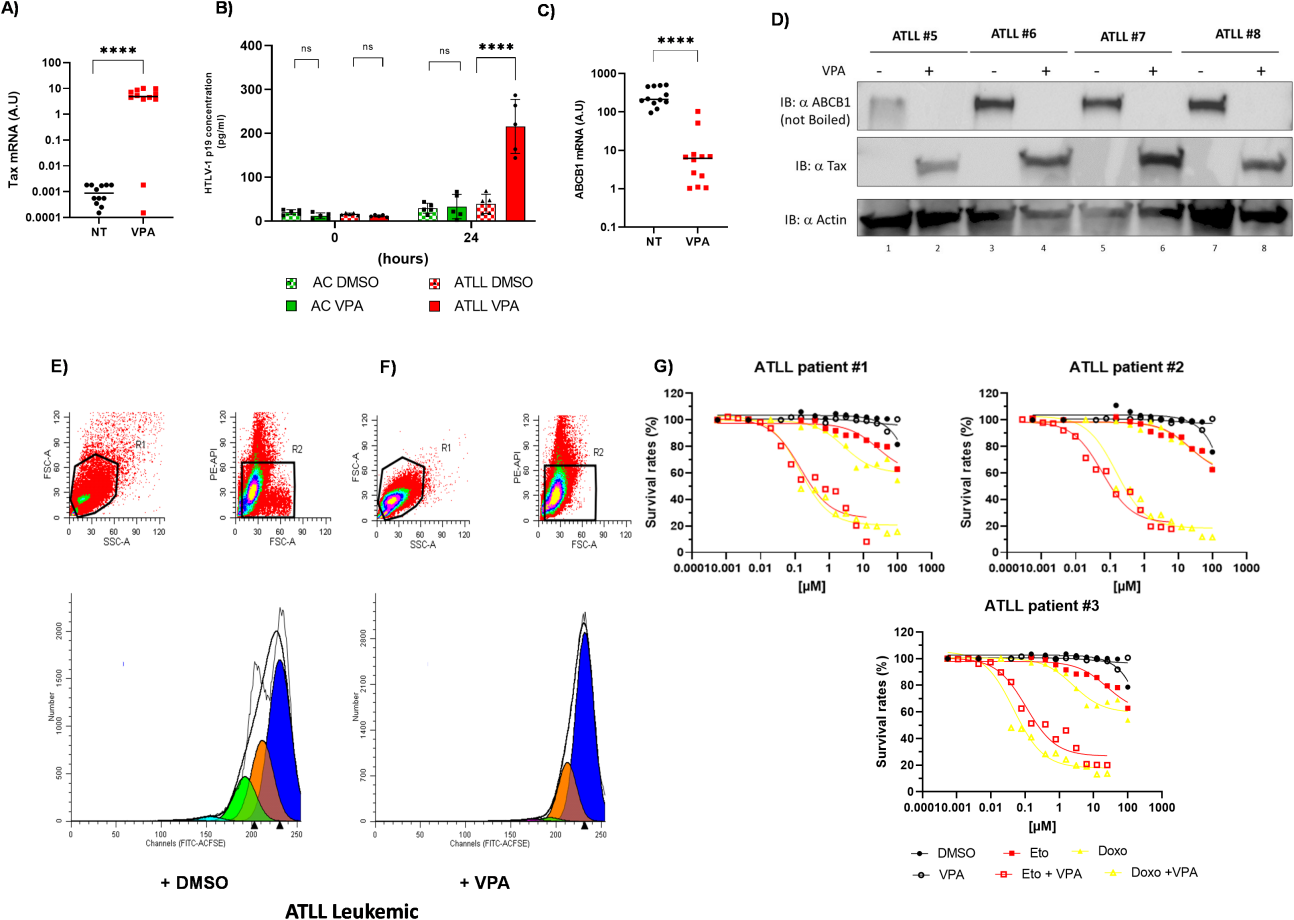
Valproic acid enhances the effects of doxorubicin and etoposide in CD8-depleted PBMCs from patients with acute ATL by reducing ABCB1 expression. **(A)** CD8^+^-depleted PBMCs from twelve acute ATLL patients were treated with 5 mM VPA for 5 days, and Tax mRNA levels were measured by RT-qPCR. **(B)** p19 Gag production after VPA treatment was assessed by ELISA in the supernatant of CD8-depleted PBMCs from five acute ATLL patients at 96 hours post-treatment (error bars indicate standard deviation). **(C)** ABCB1 mRNA levels post-VPA treatment were determined by quantitative RT-PCR and normalized to HPRT RNA levels. **(D)** ABCB1 and Tax protein levels in VPA-treated CD8-depleted PBMCs from four ATLL patients were analyzed by Western blot, with actin as a loading control. **(E, F)** VPA reduces proliferation in ATLL leukemic cells. A CFSE-based proliferation assay was performed on CD8+ depleted PBMCs from four ATLL patients. CFSE was added on day 0, and cells were harvested on day 5. Live lymphocytes were gated using FSC/SSC and FSC/PI (upper panel), and proliferation was modeled with ModFit software (lower panel). A representative example (ATL patient number #7) is shown, comparing cells cultured without VPA **(E)** or with 5 mM VPA **(F, G)** CD8+ cell–depleted PBMCs from three patients with acute ATLL were incubated with 5 mM VPA for 24 hours, followed by 48 hours of treatment with increasing concentrations of etoposide or doxorubicin. Cell viability was assessed using PrestoBlue vital dye (Invitrogen), and survival rates were calculated relative to untreated controls.

In a previous study, we showed that treatment with sodium valproate (VPA) increases Tax expression while reducing HBZ expression in HTLV-1-infected cells from asymptomatic carriers (ACs) and patients with HAM/TSP (12, 54). VPA is safe at therapeutic doses in clinical trials, including for HTLV-1-infected patients with HAM/TSP (54). Mechanistically, VPA inhibits both histone deacetylation and DNA methylation (55). Here, we examined the effects of VPA combined with etoposide or doxorubicin on primary ATLL cells. To evaluate VPA’s impact on ATLL cells, we tracked the expression patterns of tax, gag, and hbz during the culture of CD8+-cell–depleted PBMCs from six ATLL patients with leukemic subtypes and six HTLV-1 carriers without malignancy (**Supplementary Figure 4**). In HTLV-1 asymptomatic carriers (ACs), we found low levels of Tax and Gag mRNA, while HBZ expression was barely detectable even on day 5 (**Supplementary Figure 4A**). Conversely, in ATLL cells, HBZ mRNA was consistently detectable and stabilized after day 2, whereas Tax and Gag mRNA remained undetectable throughout the five-day culture (**Supplementary Figure 4B**). VPA treatment significantly increased Tax and Gag mRNA levels (Mann-Whitney U test, P < 0.05) in HTLV-1 non-leukemic cells (**Supplementary Figure 4C**), consistent with our earlier findings (12), as well as in ATLL cells (**Supplementary Figure 4D**). On the other hand, HBZ expression was notably reduced in ATLL cells following VPA treatment (**Supplementary Figure 4D**). Tax mRNA levels were significantly higher in VPA-treated ATLL samples compared to untreated controls (Mann-Whitney U test, P = 0.007) (**Figure 8A**). Since the tax gene in ATLL patients often contains deletions, insertions, nonsense mutations, or stop codons (56–59), we performed an HTLV-1 p19 gag ELISA to evaluate its transcriptional activity. We found that the Tax protein induced by VPA in our acute ATLL patients is fully functional (**Figure 8B**). Meanwhile, we saw a significant reduction in ABCB1 mRNA expression in leukemic samples treated with VPA, reaching levels nearly identical to those in non-leukemic samples (**Figure 8C** versus **Figure 3B**). We also assessed the effect of VPA on ABCB1 protein levels in PBMCs from four acute ATLL patients using WB analysis. In all four cases, VPA treatment markedly stimulated Tax protein expression and inhibited ABCB1 protein expression (**Figure 8D**).

### 3.6 VPA inhibits proliferation and enhances chemosensitivity in ATLL cells

**Figure 9:**
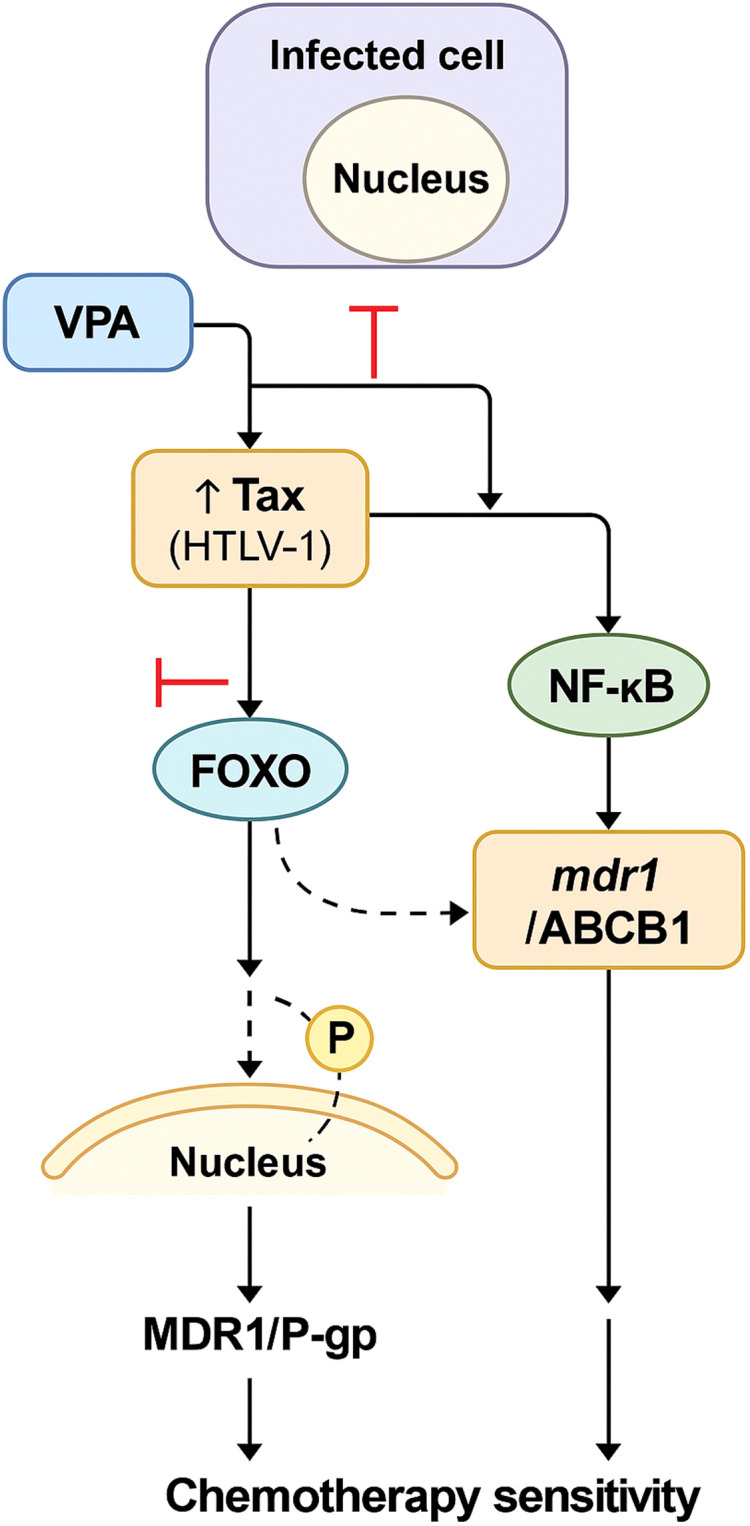
Summary of the mechanism of action of Tax in relieving ATLL chemoresistance.

Next, we assessed the proliferation of ATLL leukemic cells by staining CD8+ cell–depleted PBMCs from patients with leukemia or lymphoma using CFSE (**Figure 8E**, **Supplementary Figure 4**). As shown in **Figures 9A, B**, treatment with 5 mM, VPA significantly inhibited ATLL cell proliferation. On day 5 of culture, the average proliferation index of ATLL leukemic cells was 1.5 ± 0.2 under control conditions and 1.3 ± 0.2 following treatment with 5 mM of VPA (Wilcoxon signed-rank test, *P* = 0.03). The mean percentage of non-proliferative cells was 74.12% in controls and 90.7% in the VPA-treated group (Wilcoxon signed-rank test, *P* = 0.03; **Figures 8E, F**, **Supplementary Figure 4**). We next compared the potency and combined efficacy and potency (area under the curve; AUC) of doxorubicin and etoposide on CD8+ cell–depleted PBMCs from three patients with acute ATLL, with or without VPA pretreatment (**Figure 8G**). Pretreatment with 1 mM VPA for 24 hours significantly enhanced the potency of both Eto and Doxo in these cells (**Figure 8G**, **Table 2**).

**Table 2:** Potency and the combined efficacy of chemotherapeutic agents against primary CD8-depleted PBMCs from ATLL patients.

	Doxorubicin	Etoposide	Doxorubicin + VPA	Etoposide + VPA
Drug IC50 (µM)	>100 #	>100 #	0,04694 to 0,1357	0,1178 to 0,2684
AUC (unit)	7643 +/- 50,16	6501 + 44,45	1558 + 104,4*****	324 + 15,92*****

Primary CD8-depleted PBMCs were cultured under low-binding conditions. Data are presented as mean ± standard deviation (n = 3). Significance values: p ≤ 0.05 (*), p ≤ 0.0001 (****). IC_50_ values were estimated from raw data where not directly calculated by GraphPad Prism.

## 4 Discussion and conclusion

Adult T-cell leukemia/lymphoma (ATLL) is a highly aggressive cancer of mature T cells that develops in association with human T-cell lymphotropic virus type 1 (HTLV-1). Managing this disease remains difficult, as standard chemotherapy approaches, including CHOP (cyclophosphamide, doxorubicin, vincristine, and prednisone) and even more intensive regimens, have not significantly improved outcomes. Prognosis is especially unfavorable for patients with the acute and lymphoma subtypes (25, 39, 40). A significant obstacle to achieving durable remission and improved overall survival is the development of chemoresistance. A deeper understanding of the complex mechanisms underlying this resistance is essential for the development of more effective terapeutic strategies. Although direct evidence linking ABCB1 to chemoresistance in HTLV-1-infected cells remains scarce (22–25), analogous mechanisms described in solid tumors suggest a potential connection.

Chemoresistance in ATLL is a multifactorial process involving the overexpression of drug efflux pumps from the ATP-binding cassette (ABC) transporter superfamily, alterations in apoptotic pathways, and modulation of the bone marrow microenvironment (26, 29, 41). Among these factors, overexpression of ABC transporters such as ABCB1 (also known as P-glycoprotein [P-gp] or MDR-1) is frequently associated with resistance to a broad spectrum of chemotherapeutic agents used in ATLL treatment, including anthracyclines (e.g., doxorubicin) and epipodophyllotoxins (e.g., etoposide) (26, 29, 41). Therefore, elucidating the regulatory mechanisms governing ABCB1 expression in ATLL cells is crucial for identifying novel therapeutic targets.

ABCB1 functions as an ATP-dependent efflux pump, actively transporting a wide variety of xenobiotics and anticancer drugs out of cells (26, 29, 41). This activity reduces intracellular drug concentrations, thereby decreasing therapeutic efficacy (42). While ABCB1 is usually expressed in tissues such as the intestine, liver, kidney, and the blood-brain barrier, elevated levels in cancer cells are associated with resistance to agents including doxorubicin, vincristine, paclitaxel, and etoposide (42). In hematologic malignancies, including acute leukemias and lymphomas, increased ABCB1 expression has been linked to poorer treatment responses and reduced overall survival (43). While there’s limited direct evidence linking ABCB1/MDR1 to drug resistance in ATLL, the increased ABCB1 expression is frequently observed in ATLL patients, particularly in relapsed or refractory disease. It is likely to contribute to resistance against multiple chemotherapy agents (23). As an ATP-dependent efflux pump, ABCB1 exports drugs such as doxorubicin and cisplatin, thereby reducing intracellular drug concentrations (26, 29, 41). It has also been widely recognized as a crucial factor in the development of chemoresistance across various cancers, including bladder cancer (30), breast cancer (31), and osteosarcoma (33).

Both verapamil and CsA act as competitive substrates and inhibitors of ABCB1, competing with endogenous and cytotoxic drug substrates for overlapping binding sites (44–46). Achieving effective competition requires micromolar concentrations, which often exceed non-toxic therapeutic levels in cells. In various cell types, including T-cell malignancies, ABCB1 expression and transporter kinetics differ from those observed in classic MDR models, resulting in insufficient verapamil or CsA binding occupancy to adequately inhibit efflux (44–46). Additionally, ABCB1’s multiple drug-binding domains and conformational flexibility allow substrates and modulators to bind without impairing ATPase activity or efflux unless multiple inhibitory sites are engaged. Typically, verapamil and CsA fail to effectively target these allosteric sites (44–46). Both compounds also have off-target effects—verapamil affects L-type Ca²^+^ channels; CsA impacts calcineurin and immune pathways—that hinder the dose escalation required for effective ABCB1 inhibition (44–46). Moreover, ATLL cells often express alternative efflux transporters, such as ABCG2, or utilize uncharacterized efflux systems unaffected by verapamil or CsA, further diminishing the impact of ABCB1 blockade alone. High ABCB1 expression and altered membrane lipid composition in ATLL cells can also impair drug-transporter interactions, thereby decreasing the efficacy of competitive inhibitors. Consequently, the limitations of verapamil and CsA as ABCB1 inhibitors in ATLL stem from their low binding affinity at therapeutic doses, competitive interactions, and cellular environments in which modulating ABCB1 alone cannot sufficiently alter drug efflux. These observations underscore that, despite the source of the inhibitors, unpredictable pharmacokinetic drug interactions, the involvement of multiple drug transporters in ATLL cells, and variability in transporter expression among individuals, these factors remain significant barriers to effectively restoring drug sensitivity in the clinic. Recognizing the limited success of traditional inhibitors in overcoming multidrug resistance, we have developed an innovative approach to bypass ABC transporters by targeting ABCB1 mRNA.

The viral oncoprotein Tax plays a key role in HTLV-1 pathogenesis and may also contribute to chemoresistance (47, 48). Tax is a potent transcriptional activator that disrupts multiple cellular pathways, notably the NF-κB signaling cascade (49). Persistent NF-κB activation is characteristic of HTLV-1-infected cells and is implicated in various aspects of ATLL development and progression, including cell survival and proliferation (49). Chuang et al. reported that Tax may upregulate ABCB1 expression in Vero cells, potentially via activation of HIF-1α, and subsequent indirect upregulation of ABCB1 (47). However, the precise mechanisms governing ABCB1 regulation in ATLL remain to be elucidated. In this study, we proposed a novel role for Tax in modulating chemoresistance by regulating the expression of drug efflux pumps. We found that Tax, via activation of the canonical NF-κB pathway, downregulated ABCB1 expression in HuT78 and JPX9 T cells, as well as in CD8^+^-depleted PBMCs from patients with acute ATLL. The mechanisms by which Tax-activated NF-κB reduces ABCB1 levels are likely complex (**Figure 9**). As a transcription factor, NF-κB regulates the expression of numerous genes, and its activation can result in diverse downstream effects (17). It’s possible that NF-κB activation by Tax indirectly suppresses ABCB1 transcription by inducing other regulatory factors or epigenetic modifications. Several studies indicated that FoxO transcription factors can enhance ABCB1 expression, thereby contributing to drug resistance (50). For example, in K562 leukemic cells, doxorubicin has been shown to activate FoxO3a (51). FoxO3a binds to the ABCB1 promoter and increases its transcription, thereby increasing ABCB1 levels and conferring multidrug resistance. The Tax protein has been shown to inhibit FoxO proteins through multiple mechanisms (52, 53). Specifically, Tax promotes the ubiquitination and proteasomal degradation of FoxO4, thereby reducing its levels and activity. Another possibility is that since Tax activates the PI3K/Akt pathway, it may lead to phosphorylation of FoxO proteins, promoting their nuclear export and subsequent inactivation (54, 55). Alternatively, Tax may influence post-transcriptional or post-translational mechanisms that affect ABCB1 function or stability. Further studies are required to elucidate the precise molecular pathways underlying Tax-mediated downregulation of ABCB1.

Given the challenges posed by chemoresistance in ATLL, there is growing interest in strategies to overcome this barrier and enhance the efficacy of existing chemotherapeutic agents. Epigenetic modulators, such as histone deacetylase inhibitors (HDACi), have emerged as promising therapeutic options. However, preclinical studies investigating epigenetic drugs in ATLL remain limited. The HDAC inhibitor depsipeptide (romidepsin) has been shown to induce growth arrest and cell death in a murine model of human ATL (56). Other HDAC inhibitors, including MS-275, suberoylanilide hydroxamic acid (SAHA), and LBH589 (panobinostat), have been reported to inhibit proliferation, arrest the cell cycle at the G2/M phase, and induce apoptosis in freshly isolated ATL cells (57). Valproic acid (VPA), a short-chain fatty acid, has demonstrated anti-tumor activity in various hematologic malignancy cell lines—including ATLL—by promoting apoptosis (12, 58–60). In parallel, AR-42, a novel HDAC inhibitor, has been shown to prolong survival in ATLL-engrafted mice (61). HDAC inhibitors such as vorinostat, pralatrexate, and romidepsin have been successfully used and approved for the treatment of T-cell lymphomas (62). Several clinical trials are currently investigating the use of VPA in different cancer types (63–66). Notably, a two-year clinical trial in patients with HAM/TSP concluded that VPA does not durably modify HTLV-1 proviral load or alleviate neurological symptoms (67). Additionally, a preliminary study investigated the use of VPA as consolidation therapy after discontinuation of AZT-IFN treatment in ATLL patients (68). One clinical trial is currently evaluating the effect of VPA therapy on persistent clonal disease during complete or stable partial remission (Clinicaltrials.gov: NCT00079378). The fact that VPA is listed in the pharmacopeia and carries minimal risk of clinically relevant toxicity supports its continued clinical investigation in HTLV-1-associated diseases. However, this area of research remains in need of a stronger evidence base.

The potential use of epigenetic drugs such as valproate to enhance the efficacy of conventional chemotherapy in ATLL is promising. VPA may counteract additional resistance mechanisms beyond ABCB1 overexpression, potentially reversing or bypassing chemoresistance by modulating gene expression patterns in ATLL cells. For example, VPA could upregulate pro-apoptotic genes or downregulate anti-apoptotic factors, thereby increasing the susceptibility of ATLL cells to the cytotoxic agents such as etoposide and doxorubicin. Consistent with this, our data demonstrate that valproate significantly enhances the cytotoxicity of these drugs in patient-derived ATLL PBMCs. Two principal mechanisms may underlie this increased potency. First, VPA relieves the epigenetic suppression of the HTLV-1 5’LTR, allowing the re-expression of Tax in CD8+-depleted PBMCs from patients with acute ATLL, and leading to the downregulation of ABCB1. Second, our findings indicate that ABCB1 expression decreases upon Tax expression. Additionally, VPA may increase cellular susceptibility to the DNA-damaging effects of chemotherapeutic agents, potentially by modulating pathways involved in DNA repair, cell cycle regulation, or apoptosis (65, 69).

These findings have important implications for the treatment of acute ATLL. The observation that Tax may downregulate ABCB1 via NF-κB adds complexity to the understanding of chemoresistance in this disease. Our findings were also supported by the downregulation of ABCB1 by the valproate treatment. Indeed, Valproate has also been shown to reduce the activity of the NF-κB pathway, although this effect depends on the cell type, dose, and experimental conditions (70–76). Although reduced ABCB1 levels could, in theory, enhance drug sensitivity, the aggressive biology and multiple resistance mechanisms characteristic of acute ATLL often limit this effect. Therefore, therapeutic strategies targeting Tax or NF-κB alone are unlikely to be sufficient to overcome chemoresistance in this context. Our data demonstrating the synergistic effects of valproate in combination with etoposide and doxorubicin in primary ATLL patient samples provides a strong rationale for testing such combination therapies. Epigenetic agents such as valproate may represent a promising strategy to sensitize ATLL cells to conventional chemotherapy and potentially improve treatment outcomes. Future clinical trials combining HDAC inhibitors with standard chemotherapeutic regimens will be essential to validate these preliminary findings.

In conclusion, elucidating the complex mechanisms underlying chemoresistance in ATLL is essential for the development of more effective therapies. Our findings highlight the intricate role of Tax in potentially downregulating ABCB1 expression via NF-κB signaling, although additional factors are likely to contribute to drug resistance in acute ATLL. Notably, our data strongly support the use of the epigenetic modulator valproate to enhance the efficacy of etoposide and doxorubicin in primary ATLL patient cells. These results highlight the therapeutic potential of combining epigenetic agents with standard chemotherapy to overcome chemoresistance in this challenging hematologic malignancy. Continued research into targeted therapies, immunomodulatory strategies, and resistance-reversal approaches will be critical to improving the currently poor outcomes for patients with this rare but aggressive disease.

Resistance in ATLL is multifactorial, involving ABCB1-mediated drug efflux, dysregulated apoptosis (e.g., BCL-2, PI3K/AKT), constitutive NF-κB/MAPK signaling, and epigenetic silencing. Our findings indicate that a Tax-based “shock-and-kill” strategy may be a viable approach to overcoming chemoresistance in ATLL, while recognizing that its clinical translation would require stringent controls and further validation. In this framework, the “shock” component involves the transient reactivation of the HTLV-1 viral protein Tax, which, in our study, was associated with reduced ABCB1 expression and a concomitant restoration of sensitivity to chemotherapeutic agents such as etoposide and doxorubicin. Pharmacological modulation using histone deacetylase inhibitors, including valproic acid, which are already approved for clinical use, may provide a feasible means of inducing short-term Tax expression. Importantly, this approach does not aim to sustain viral gene expression or enhance viral replication, but instead seeks to exploit a limited therapeutic window in which drug efflux mechanisms are attenuated, thereby increasing intracellular drug accumulation. The “kill” phase would then be mediated by conventional chemotherapy, rather than by Tax itself. Nevertheless, several challenges must be addressed, including the potential oncogenic or immunomodulatory effects of Tax, the requirement for precise temporal regulation of its expression, and the risk of viral reactivation. Future studies in relevant *in vivo* models and well-designed clinical investigations will be necessary to determine the safety, therapeutic benefit, and optimal integration of transient Tax induction into combination treatment strategies for ATLL.

## Data Availability

The datasets generated and/or analyzed during the current study are available from the corresponding author upon reasonable request. All relevant data supporting the findings of this study are included in the article and its **Supplementary Material**.

## Supplementary material

Supplementary Figure 1 Expression analysis of solute carrier transporters, glutathione S-transferases, and LRP1 in HTLV-1-derived cell lines. **(A–E)** Expression of six genes—*slco2b1, slc22a4, slc22a5, gstp, gstt1*, and *lrp1*—associated with chemoresistance in lymphoma, was assessed in two HTLV-1-negative cell lines (HuT78, Jurkat) and three HTLV-1-derived cell lines (HuT102, C81–66, ATL-2) by RT-qPCR. Statistical significance was determined using a one-way ANOVA with Dunn’s multiple comparisons post-test: ns, p ≤ 0.05, ** p ≤ 0.01, *** p ≤ 0.001, **** p ≤ 0.0001.

Supplementary Figure 2 Expression analysis of solute carrier transporters, glutathione S-transferases, and LRP1 in PBMCs from HTLV-1-infected patients. **(A–E)** Expression of six genes—*slco2b1, slc22a4, slc22a5, gstp1, gstt1*, and *lrp1*—associated with chemoresistance in lymphoma was evaluated in PBMCs from five uninfected donors (CD4+ NI), twelve HTLV-1 asymptomatic carriers (AC), and thirteen untreated acute ATLL patients (ATLL) by RT-qPCR. Statistical significance was determined using a one-way ANOVA with Dunn’s multiple comparisons post-test: ns, p ≤ 0.05, ** p ≤ 0.01, *** p ≤ 0.001, **** p ≤ 0.0001.

Supplementary Figure 3 Surface ABCB1 expression is decreased in cells expressing Tax. HEK293T cells were transfected with the Tax-Flag plasmid. After 48 hours, cells were fixed and stained with an anti-ABCB1 antibody, and surface ABCB1 protein levels were assessed by FACS analysis.

Supplementary Figure 4 Valproate effects on Tax, Gag, and HBZ expression in HTLV-1–infected nonmalignant and ATLL cells. Tax (red), Gag (blue), and HBZ-mRNA (green) levels were measured in *ex vivo* cultured cells from HTLV-1–infected non-ATL cells (AC) and ATL leukemic (ATLL) cells, either untreated **(A)** or treated with 5-mM VPA by RT-qPCR **(B)**. Medians and first quartiles (in arbitrary units, AU) are shown for six samples in each group.

Supplementary Figure 5 VPA reduces proliferation in ATL leukemic cells. A CFSE-based proliferation assay was performed on CD8^+^depleted PBMCs from two ATL leukemic patients and one ATL lymphoma patient. CFSE was added on day 0, and cells were harvested on day 5. Live lymphocytes were gated using FSC/SSC and FSC/PI **(A, B)**, and proliferation was analyzed using ModFit software.
