# Supplementary material for: Valproate reactivates HTLV-1 tax and reduces ABCB1/MDR1 expression in PBMCs derived from ATLL patients
Source: Front Oncol. 2026 Mar 12;16:1721313. doi: 10.3389/fonc.2026.1721313 (PMC13017922; doi:10.3389/fonc.2026.1721313)
Supplement: Supplementary file 1 [file Table1.docx]

| Supplemental Table 1: Primers used for RT-qPCR | | | | | |
| --- | --- | --- | --- | --- | --- |
| Genes: |  | **Unique Assay ID:** | | **Assay Design:** | |
| ABCA3 | Biorad | qHsaCED0046511 | | Exonic | |
| ABCB1 | Biorad | qHsaCED0056970 | | Exonic | |
| ABCB5 | Biorad | qHsaCID0018521 | | Intron-spanning | |
| ABCC1 | Biorad | qHsaCID0016624 | | Intron-spanning | |
| ABCG2 | Biorad | qHsaCID0009494 | | Intron-spanning | |
| LRP1 | Biorad | qHsaCED0003623 | | Exonic | |
| GSTP1 | Biorad | qHsaCID0020557 | | Intron-spanning | |
| GSTT1 | Biorad | qHsaCED0001054 | | Exonic | |
|  |  |  |  |  |  |
| Genes: | **sequence** | | |  | |
| SLCO2B1-FW | GTACCCCAGGTACCAGACAAG | | |  |  |
| SLCO2B1-Rev | TATGGAACACACTTGGCCG | | |  |  |
| SLC22A4-Fw | GTGGGAAGCATGCGGGACTAC | | |  |  |
| SLC22A4-Rev | CCATTGAAGCCATTGGGGATG | | |  |  |
| SLC22A5-Fw | CAGAGGTGATCATCCGCAAG | | |  |  |
| SLC22A5-Rev | GGACTGCTGCTTCTTGGAAC | | |  |  |
| SLC47A1-Fw | CATCGTGTATGAACTGGCCATC | | |  |  |
| SLC47A1-Rev | GAGCGTTTCCTACCCGGAC | | |  |  |
| HPRT -FW | AAGGGCATATCCTACAACAAAC | | |  |  |
| HPRT -Rev | GGTCAGGCAGTATAATCCAAAG | | |  |  |
| Tax-FW | ACCAACACCATGGCCCA | | |  |  |
| Tax-Rev | GAGTCGAGGGATAAGGAAC | | |  |  |
| HBZ-Fw | ATGGCGGCCTCACCGTCGCAG | | |  |  |
| HBZ-Rev | GGTCAGGCAGTATAATCCAAAG | | |  |  |
