# Supplementary figures and images for: Valproate reactivates HTLV-1 tax and reduces ABCB1/MDR1 expression in PBMCs derived from ATLL patients
Source: Front Oncol. 2026 Mar 12;16:1721313. doi: 10.3389/fonc.2026.1721313 (PMC13017922; doi:10.3389/fonc.2026.1721313)

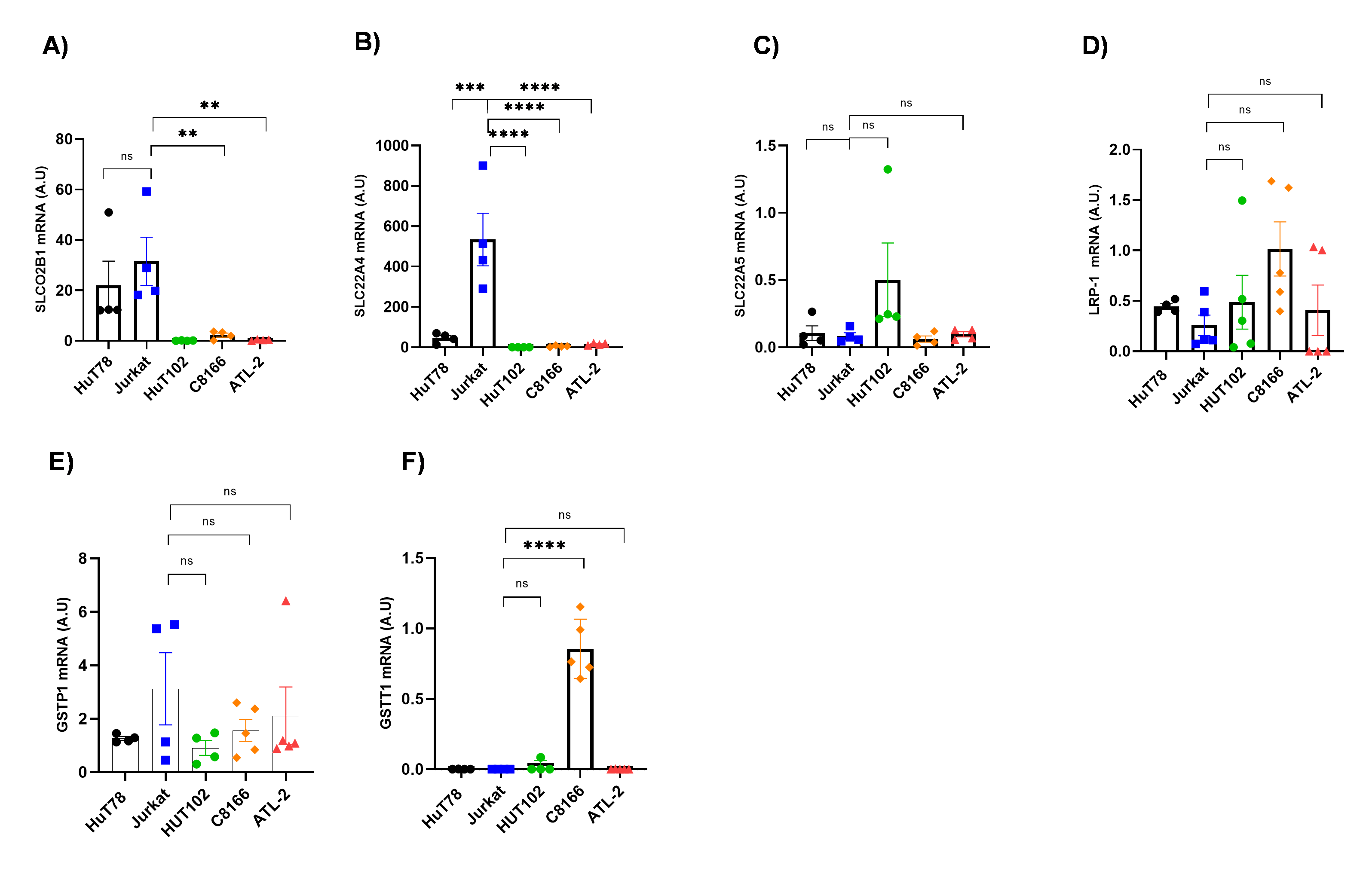

Supplement: Supplementary Figure 1 — Expression analysis of solute carrier transporters, glutathione S-transferases, and LRP1 in HTLV-1-derived cell lines. (A–E) Expression of six genes—slco2b1, slc22a4, slc22a5, gstp, gstt1, and lrp1—associated with chemoresistance in lymphoma, was assessed in two HTLV-1-negative cell lines (HuT78, Jurkat) and three HTLV-1-derived cell lines (HuT102, C81–66, ATL-2) by RT-qPCR. Statistical significance was determined using a one-way ANOVA with Dunn’s multiple comparisons post-test: ns, p ≤ 0.05, ** p ≤ 0.01, *** p ≤ 0.001, **** p ≤ 0.0001. [file Image1.tif]

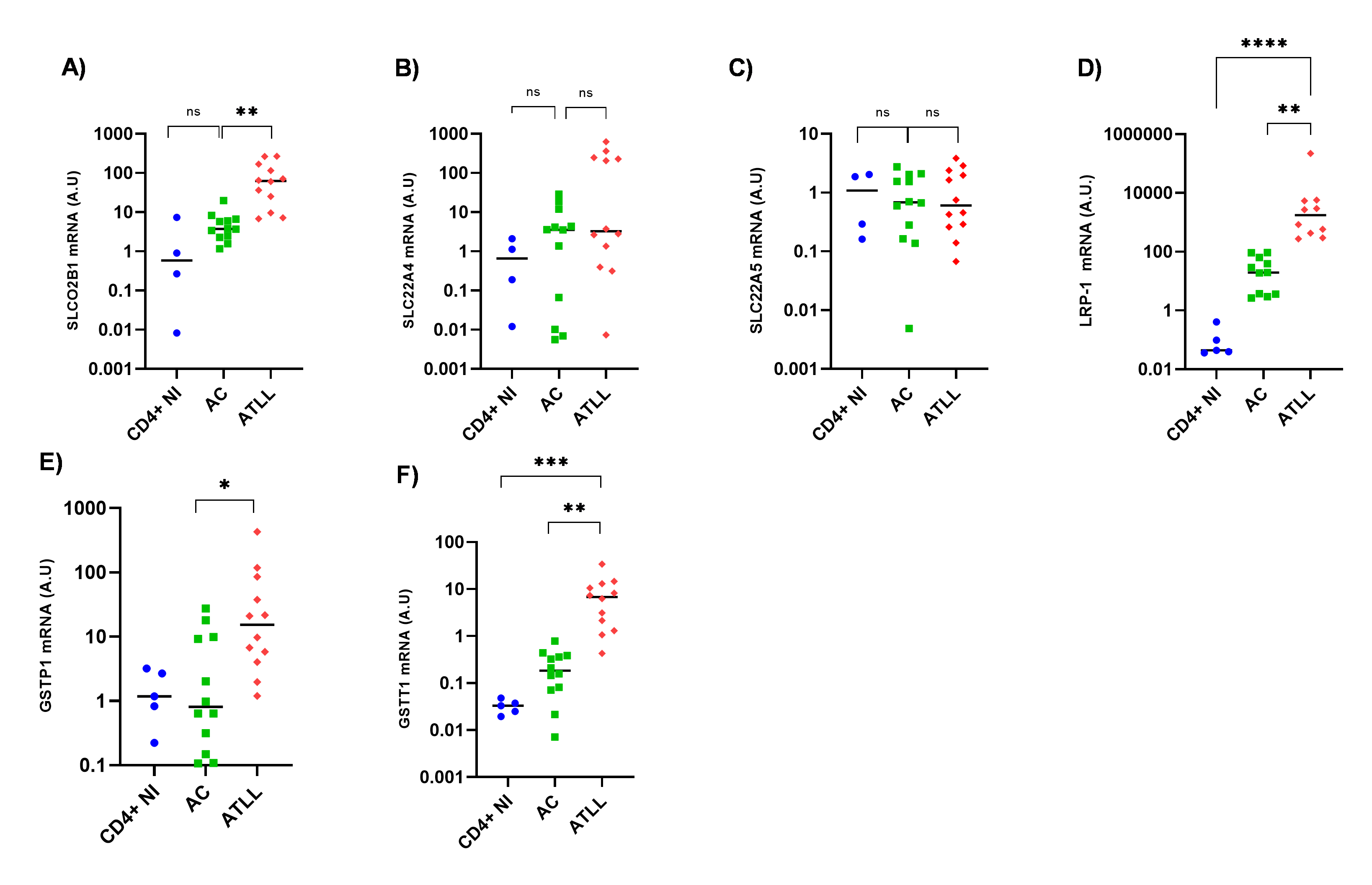

Supplement: Supplementary Figure 2 — Expression analysis of solute carrier transporters, glutathione S-transferases, and LRP1 in PBMCs from HTLV-1-infected patients. (A–E) Expression of six genes—slco2b1, slc22a4, slc22a5, gstp1, gstt1, and lrp1—associated with chemoresistance in lymphoma was evaluated in PBMCs from five uninfected donors (CD4+ NI), twelve HTLV-1 asymptomatic carriers (AC), and thirteen untreated acute ATLL patients (ATLL) by RT-qPCR. Statistical significance was determined using a one-way ANOVA with Dunn’s multiple comparisons post-test: ns, p ≤ 0.05, ** p ≤ 0.01, *** p ≤ 0.001, **** p ≤ 0.0001. [file Image2.tif]

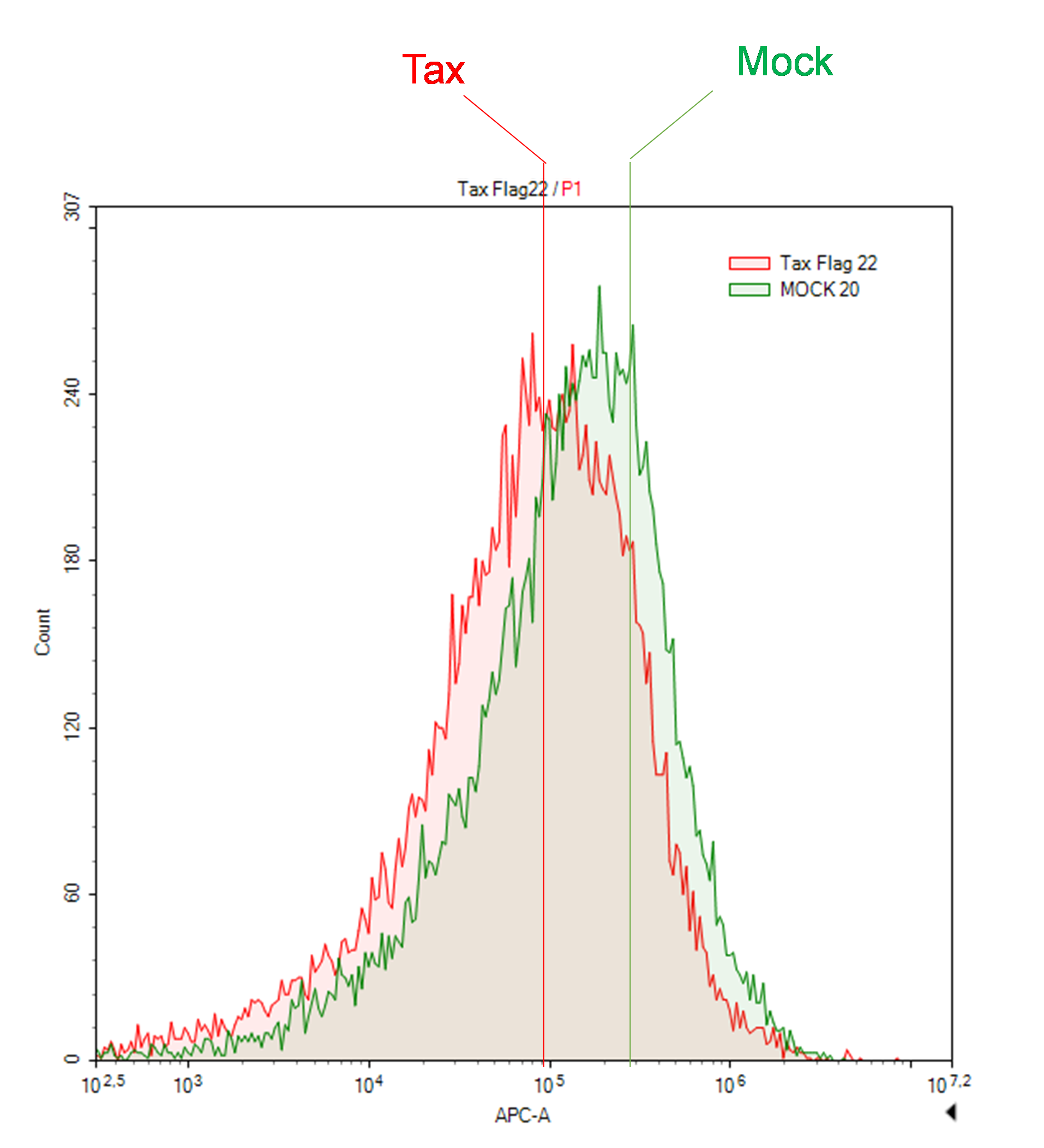

Supplement: Supplementary Figure 3 — Surface ABCB1 expression is decreased in cells expressing Tax. HEK293T cells were transfected with the Tax-Flag plasmid. After 48 hours, cells were fixed and stained with an anti-ABCB1 antibody, and surface ABCB1 protein levels were assessed by FACS analysis. [file Image3.tif]

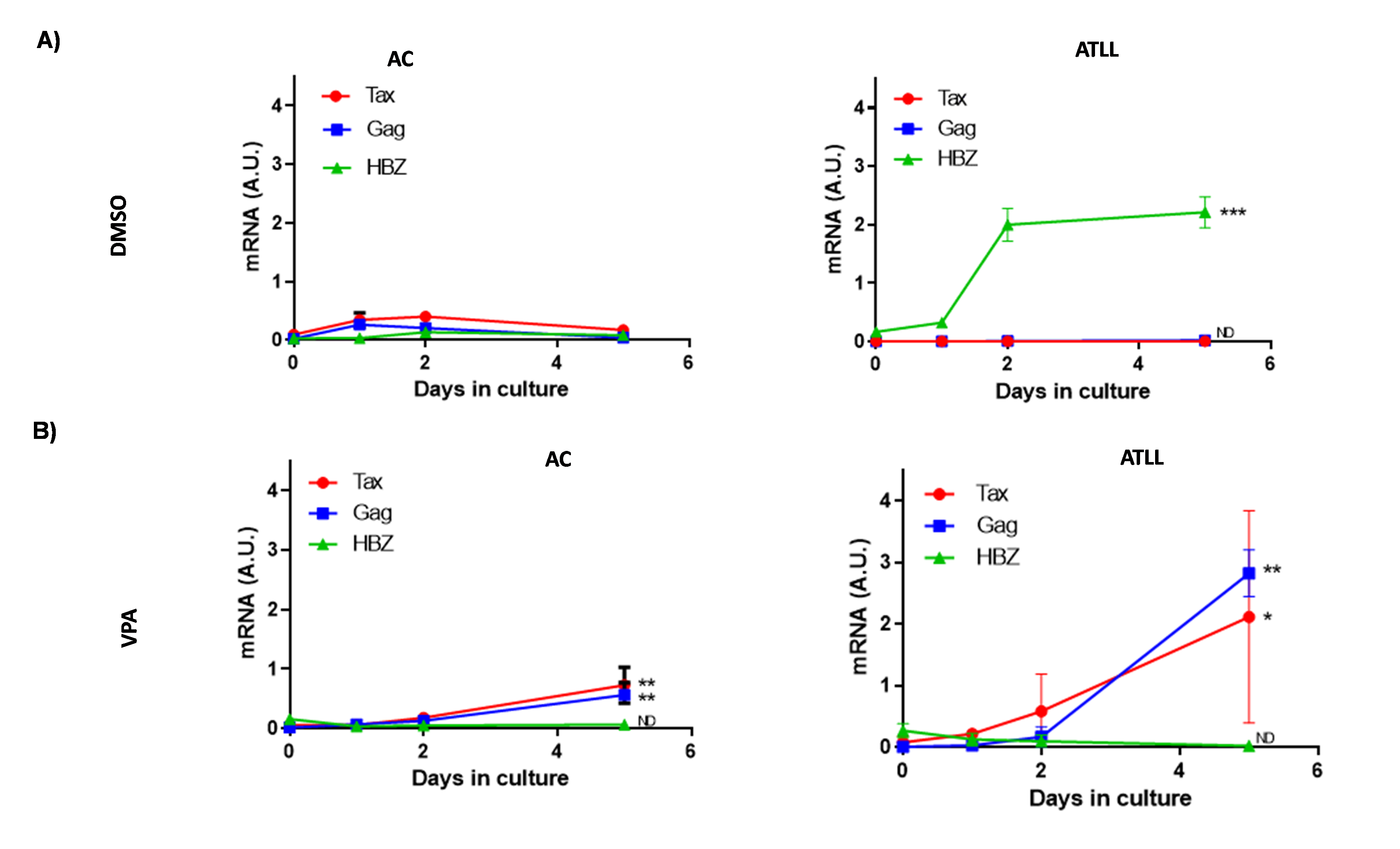

Supplement: Supplementary Figure 4 — Valproate effects on Tax, Gag, and HBZ expression in HTLV-1–infected nonmalignant and ATLL cells. Tax (red), Gag (blue), and HBZ-mRNA (green) levels were measured in ex vivo cultured cells from HTLV-1–infected non-ATL cells (AC) and ATL leukemic (ATLL) cells, either untreated (A) or treated with 5-mM VPA by RT-qPCR (B). Medians and first quartiles (in arbitrary units, AU) are shown for six samples in each group. [file Image4.tif]

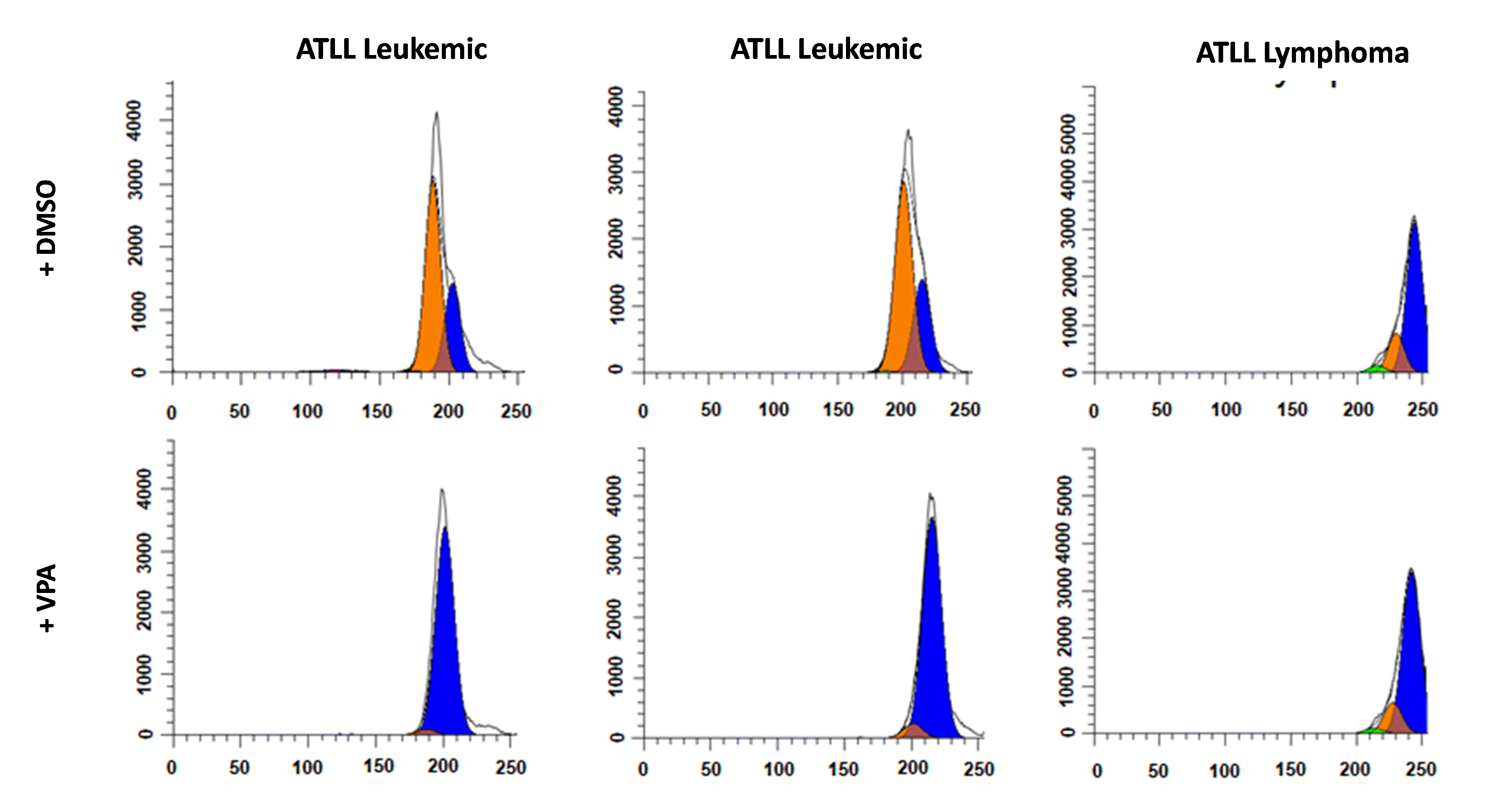

Supplement: Supplementary Figure 5 — VPA reduces proliferation in ATL leukemic cells. A CFSE-based proliferation assay was performed on CD8+depleted PBMCs from two ATL leukemic patients and one ATL lymphoma patient. CFSE was added on day 0, and cells were harvested on day 5. Live lymphocytes were gated using FSC/SSC and FSC/PI (A, B), and proliferation was analyzed using ModFit software. [file Image5.tif]
